# High-entropy-alloy nanoparticles with 21 ultra-mixed elements for efficient photothermal conversion

**DOI:** 10.1093/nsr/nwac041

**Published:** 2022-03-04

**Authors:** Yijun Liao, Yixing Li, Rongzhi Zhao, Jian Zhang, Lizhong Zhao, Lianze Ji, Zhengyu Zhang, Xiaolian Liu, Gaowu Qin, Xuefeng Zhang

**Affiliations:** Key Laboratory for Anisotropy and Texture of Materials (MOE), School of Materials Science and Engineering, Northeastern University, Shenyang 110819, China; Key Laboratory for Anisotropy and Texture of Materials (MOE), School of Materials Science and Engineering, Northeastern University, Shenyang 110819, China; Key Laboratory for Anisotropy and Texture of Materials (MOE), School of Materials Science and Engineering, Northeastern University, Shenyang 110819, China; Institute of Advanced Magnetic Materials, College of Materials and Environmental Engineering, Hangzhou Dianzi University, Hangzhou 310012, China; Institute of Advanced Magnetic Materials, College of Materials and Environmental Engineering, Hangzhou Dianzi University, Hangzhou 310012, China; Institute of Advanced Magnetic Materials, College of Materials and Environmental Engineering, Hangzhou Dianzi University, Hangzhou 310012, China; Key Laboratory for Anisotropy and Texture of Materials (MOE), School of Materials Science and Engineering, Northeastern University, Shenyang 110819, China; Institute of Advanced Magnetic Materials, College of Materials and Environmental Engineering, Hangzhou Dianzi University, Hangzhou 310012, China; Key Laboratory for Anisotropy and Texture of Materials (MOE), School of Materials Science and Engineering, Northeastern University, Shenyang 110819, China; Institute of Advanced Magnetic Materials, College of Materials and Environmental Engineering, Hangzhou Dianzi University, Hangzhou 310012, China; Key Laboratory for Anisotropy and Texture of Materials (MOE), School of Materials Science and Engineering, Northeastern University, Shenyang 110819, China; Key Laboratory for Anisotropy and Texture of Materials (MOE), School of Materials Science and Engineering, Northeastern University, Shenyang 110819, China; Institute of Advanced Magnetic Materials, College of Materials and Environmental Engineering, Hangzhou Dianzi University, Hangzhou 310012, China

**Keywords:** high-entropy alloy, nanoparticle, ultra-mixing, arc-discharged method, photothermal conversion

## Abstract

Multi-metallic nanoparticles have been proven to be an efficient photothermal conversion material, for which the optical absorption can be broadened through the interband transitions (IBTs), but it remains a challenge due to the strong immiscibility among the repelling combinations. Here, assisted by an extremely high evaporation temperature, ultra-fast cooling and vapor-pressure strategy, the arc-discharged plasma method was employed to synthesize ultra-mixed multi-metallic nanoparticles composed of 21 elements (FeCoNiCrYTiVCuAlNbMoTaWZnCdPbBiAgInMnSn), in which the strongly repelling combinations were uniformly distributed. Due to the reinforced lattice distortion effect and excellent IBTs, the nanoparticles can realize an average absorption of >92% in the entire solar spectrum (250 to 2500 nm). In particular, the 21-element nanoparticles achieve a considerably high solar steam efficiency of nearly 99% under one solar irradiation, with a water evaporation rate of 2.42 kg m^–2^ h^–1^, demonstrating a highly efficient photothermal conversion performance. The present approach creates a new strategy for uniformly mixing multi-metallic elements for exploring their unknown properties and various applications.

## Teaser

A record 21-element high-entropy-alloy nanoparticle with uniformly elemental distribution is synthesized via an arc-discharged plasma method assisted with the vapor pressure design strategy, in which an excellent photothermal conversion performance at the UV-Vis-IR region can be realized.

## INTRODUCTION

Photothermal conversion materials have been attracting considerable attention with regard to their water evaporation applications, as a result of the intensified consumption of clean water and severe pollution of water resources recently [1–5]. To achieve a satisfying photothermal conversion efficiency, the essential condition is to absorb solar energy with wavelengths of between 250 to 2500 nm as much as possible [6–8]. Consequently, a series of mechanisms, such as the plasmonic heating of metallic nanoparticles (e.g. Au [6], Ag [9] and Al [7]), non-radiative relaxation of semiconductors (e.g. copper selenide nanocrystals [10]) and thermal vibration of molecules [11], have been widely investigated. Despite the achievement of photothermal conversion performance, the aforementioned materials still lack the capacity to cover the entire solar spectrum, thus limiting the optimization of optical absorption and solar-driven water evaporation behavior [1,2,6,7,11].

In our recent research, it has been proposed that multi-metallic nanoparticles, so-called high-entropy-alloy nanoparticles (HEA-NPs) [12–15], are one of the most promising candidates for tailoring the optical absorption property [16,17]. Such a performance is induced by the reinforced interband transitions (IBTs), contributed by the fully filled energy regions around the Fermi level [12,18]. Therefore, the solar energy absorption property can be optimized through the addition of composited elements [17], resulting in a broadened working bandwidth and thus improving the photothermal conversion performance. However, when the composited elements exceed 3*d* transition metals, the related research for understanding the relationship between the HEA-NPs and photothermal conversion performance is still lacking, and the crucial issue is how to cross the immiscibility of composited elements to synthesize the HEAs [19]. Meanwhile, it has been found that phase separation has occurred when elements exceed eight in our previous experiments. Thus, it is necessary to improve our synthesized method and meanwhile explore the limits of the method. Surprisingly, it has been reported that the HEA-NPs composed of 15 elements with strongly repelling combinations were synthesized through a carbon-thermal shock method, and the immiscibility can be overcome by the high-entropy effect and non-equilibrium synthesized process [20,21], promising a pathway for breaking the restrictions of elemental combination with strong repellency.

Herein, assisted by non-equilibrium synthesized conditions including extremely high evaporation temperature and ultra-fast cooling rate, we synthesized a series of HEA-NPs including 9, 13, 17 and 21 composited elements (denoted 9-, 13-, 17- and 21-HEA-NPs) through an arc-discharged method with vapor-pressure (*V.P.*) strategy. It has been found that the *V.P.* design could counterbalance the evaporation rates of composited elements, and plays a critical role in realizing the HEAs from the immiscible combinations. Meanwhile, the photothermal conversion performance can be improved along with the addition of composited elements. As a result, 21-HEA-NPs exhibit an excellent solar-steam-generation performance with nearly 99% solar steam efficiency and 2.42 kg m^–2^ h^–1^ water evaporation rate under 1 sun irradiation (1.0 kW m^–2^). The present work opens a vast space for synthesizing ultra-mixing HEA-NPs with immiscible elements, which broadens the family of HEA-NPs and provides a great platform for exploring the application of HEA-NPs.

## RESULTS AND DISCUSSION

### Fabrication and microstructure characterizations of HEA-NPs

**Figure 1.**
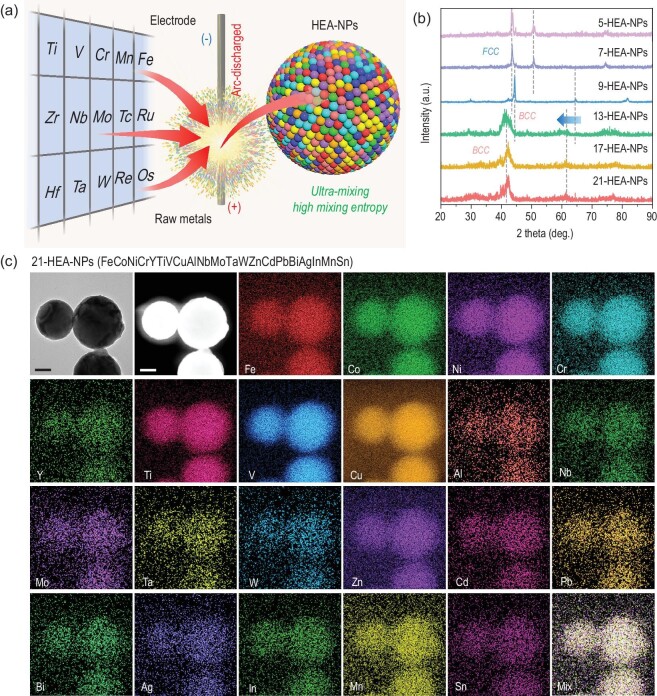
Schematic illustration and microstructure characterizations of HEA-NPs. (a) Schematic of the arc-discharged approach for synthesizing different HEA-NPs. (b) XRD patterns of the HEA-NPs with different composited elements. (c) TEM-EDS maps of 21-HEA-NPs (FeCoNiCrYTiVCuAlNbMoTaWZnCdPbBiAgInMnSn), scale bars: 50 nm.

**Figure 2.**
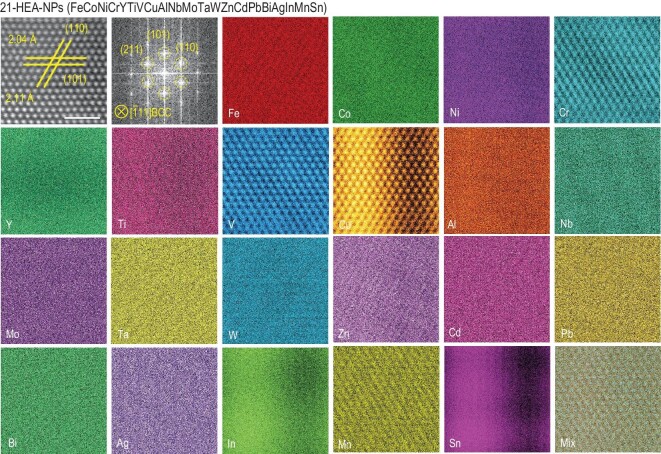
High-resolution TEM image, fast Fourier transform (FFT) image and the corresponding atomic TEM-EDS maps of 21-HEA-NPs, scale bar: 1 nm.

HEA-NPs, composed of 9, 13, 17 and 21 metallic elements, were synthesized by arc-discharging the compressed mixture of metallic powders in a mixed atmosphere of argon and hydrogen (Fig. 1a) [22,23]; the ratio of different powders was controlled via the *V.P.* strategy to realize the uniform mixing of compositions (Supporting Text in Supplementary Data) [21]. The macroscopic contents for HEA-NPs determined by inductively coupled optical emission spectroscopy (ICP-OES) (Table S1, Supplementary Data) were used to estimate the mixing entropy (Δ*S*_mix_) of HEA-NPs. The Δ*S*_mix_ of 9-, 13-, 17- and 21-HEA-NPs was calculated to be 17.83, 19.79, 21.82 and 24.28 J mol^–1^ K^–1^, respectively [17,21,24]. It was found that the mixing entropy increases synchronously with the increasing of alloying combinations. The entropy of 21-HEA-NPs was 2.69 times higher than that of 3-HEA-NPs (9.12 J mol^–1^ K^–1^), while the increased entropy could be stabilized due to the high-entropy effect [20]. The elemental distribution of 21-HEA-NPs is illustrated in Fig. 1c, using the scanning transmission electron microscope (STEM) assisted with energy-dispersive X-ray spectroscopy (EDS). Meanwhile, the high-resolution TEM image and atomic EDS maps of 21-HEA-NPs have been exhibited in Fig. 2, in which a uniform elemental distribution of most of the composited elements can be observed, while the Sn and In exhibit a slightly constituent fluctuation. As for the phase structures in Fig. 1b, the crystalline structures were changed from face-centered-cubic (FCC) to body-centered-cubic (BCC) when the composited elements exceeded nine. In addition, it can be noted that the X-ray diffraction (XRD) peaks of 13-, 17- and 21-HEA-NPs was broadened and shifted to a lower 2*θ* value, which is ascribed to the lattice distortion of the high-entropy effect and the addition of larger atomic radius elements including Ta, W, Pb and Bi [25,26].

**Figure 3.**
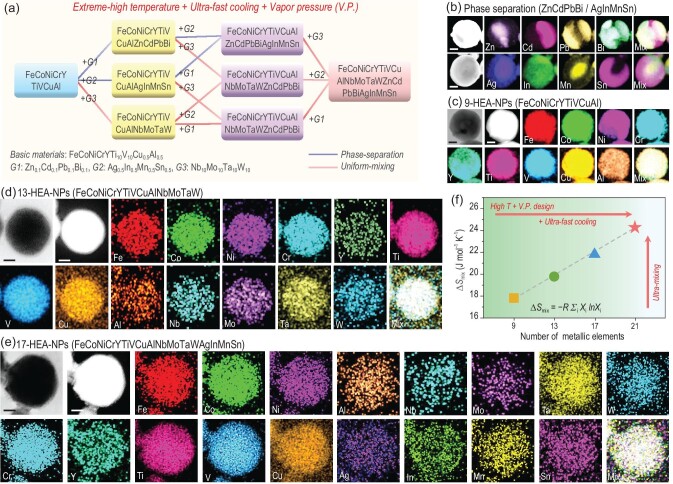
Synthetic approach design and element characterizations of HEA-NPs. (a) Flow diagram of the *V.P.* strategy and high-entropy-driven process for obtaining HEA-NPs from metals with different vapor pressure. TEM-EDS maps of (b) phase separation nanoparticles (ZnCdPbBi and AgInMnSn), (c) 9-HEA-NPs (FeCoNiCrYTiVCuAl), (d) 13-HEA-NPs (FeCoNiCrYTiVCuAlNbMoTaW) and (e) 17-HEA-NPs (FeCoNiCrYTiVCuAlNbMoTaWAgInMnSn), respectively. Scale bars: 50 nm. (f) Calculation results of the mixing entropy of different HEA-NPs based on ICP-OES results, indicating that the mixing entropy is increased along with the enhancement of composited elements.

Here, the synthesized process has been divided into three parts: extremely high evaporation temperature, ultra-fast cooling rate and *V.P.* strategy, as illustrated in Fig. 3a. According to our previous study, the high evaporation temperature created from the arc-discharged plasma can result in a non-equilibrium synthesized atmosphere, fabricating the HEA-NPs with the composited elements of Fe, Co, Ni, Ti, V, Cr and/or Cu [17]. However, it has been found that the ratio of Ti and V in the 7-HEA-NPs is relatively lower than the other composited elements. Such a phenomenon is induced by the vapor pressure of different composited elements, as exhibited in Fig. S1 (Supplementary Data), in which a low vapor pressure of Ti/V can be observed compared to other elements, indicating a slower evaporation process during the synthesized process. Therefore, to confirm the effect of the *V.P.* strategy, the nanoparticles that are composed of ZnCdPbBi, AgInMnSn and NbMoTaW were synthesized (Fig. 3b and Fig. S2, Supplementary Data). The phase separation can be observed in the former two samples through the XRD patterns, and the uniform mixing behavior appears in the NbMoTaW nanoparticles (Fig. S2, Supplementary Data).

Consequently, based on the curves of vapor pressure [21,27] (Fig. S1, Supplementary Data), different metals used in this work could be divided into three groups: (i) metals with low vapor pressure (*V.P._L_*: Mo, Nb, Ta, W), (ii) metals with medium vapor pressure (*V.P._M_*: Fe, Co, Ni, Y, Ti, V, Al, Cu, Cr, Sn), in which the Ti and V are sub-*V.P._L_* metals and Sn, Al and Cu are sub-*V.P._H_* metals; (iii) metals with high vapor pressure (*V.P._H_*: Ag, Mn, In, Pb, Bi, Zn, Cd). Following this guideline, the 9-HEA-NPs of FeCoNiCrYTiVCuAl were synthesized from the mixture with a 10-time content of Ti and V and a 0.5-time content of Cu and Al (marked as FeCoNiCrYTi_10_V_10_Cu_0.5_Al_0.5_). The corresponding synthesized rule to fabricate the HEA-NPs through the *V.P.* strategy has been summarized in the *Vapor pressure (V.P.) strategy for the preparationofHEA-NPs* section in the Supplementary Data, and it can be recognized that the optimization of chemical composition is highly correlated to the difference in vapor pressure of the composited elements. The EDS maps reveal the uniform elemental distribution of the composited elements, while the crystalline structure exhibits a combination of FCC and BCC, indicating the lattice distortion (Figs 1b and 3c). Here, using the 9-HEA-NPs as the substrate, we explore the effectiveness of the *V.P.* strategy, and a series of 13- and 17-element nanoparticles were synthesized. Uniform mixing and phase separation can be realized in different nanoparticles (Figs S10–S14, Supplementary Data), and it can be concluded that the *V.P._L_* elements are key to optimizing the evaporation rate for alloying the immiscible combinations together (Supporting Text). In addition, the 13- and 17-HEA-NPs exhibit an FCC crystalline structure with uniform distribution of composited elements, as shown in Fig. 3d and e and Figs S3 and S4 (Supplementary Data).

Furthermore, several fundamental analyses were carried out to illustrate the characters of HEA-NPs. The TEM analysis shows that all samples exhibit a sphere-like shape, and the diameter of 9-, 13-, 17- and 21-HEA-NPs was calculated to be 75, 72, 82 and 62 nm, respectively (Figs S5 and S6, Supplementary Data). It can be noted that the size of nanoparticles reduced noticeably when ultra-fast-cooling treatment was activated. The surface state of 21-HEA-NPs was characterized by X-ray photoelectron spectroscopy (XPS), and the result shows that the metals with an oxidation potential have already been oxidized under the storage process in ambient air (Figs S17–S19, Supplementary Data) [28–30].

### Optical absorption property and photothermal conversion performance of HEA-NPs

**Figure 4.**
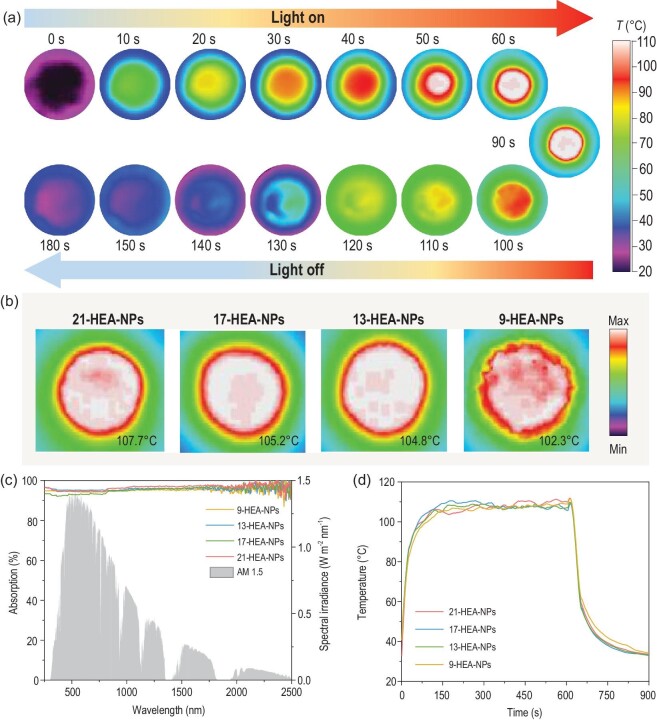
Solar harvesting property of HEA-NPs. (a) 21-HEA-NP powders under 1 solar irradiation for 90 s. (b) The maximum surface temperature of all the samples after 150 s irradiation (1 sun). (c) The solar absorption spectrum of different nanoparticles; the gray area is the solar radiation spectrum. (d) Temperature change tendency of different HEA-NPs with an irradiated time of 600 s.

The optical absorption performances of HEA-NPs have been investigated through a scattering spectrum in the wavelength region of 250 to 2500 nm (UV-Vis-NIR, ultraviolet-visible-near infrared), as shown in Fig. 4c, wherein 100 mg nanoparticles were placed in the circle area with a diameter of 24 mm (0.737 g/cm^3^). It can be recognized that, of all the samples, the 21-HEA-NPs exhibit the highest absorption property (>97%) at the NIR region, while absorption at the UV-Vis regions is reduced compared to the 9-HEA-NPs but still remains ∼95%. As for the HEAs composited with 3*d* transition metals, it has been reported that their optical absorptions at visible light can be reinforced through the addition of V and Cr, while at UV light can be enhanced through the composition of Ti and Cu [17]. Such a performance is correlated with the different 3*d* band localized energy and the *d–d* IBTs. Therefore, the absorption of entire solar spectrum can be realized in the combination of all 3*d* transition metals, but the absorption at visible light can be reduced through the addition of main group elements [31,32], which is ascribed to decreasing IBT behavior [17], and in agreement with the optical absorption property of the 21-HEA-NPs. Meanwhile, the optical absorption properties of all the HEA-NPs have been compared with carbon-based materials including graphene, graphite, carbon nanotubes and reduced graphene oxide (Table S3, ref. (S.M.) 23 to 29, Supplementary Data). It can be seen that the HEA-NPs demonstrated the best optical performance among all the materials.

Afterward, the photothermal conversion performances of all the samples were investigated in the wavelength regions of 250 to 2500 nm; 100 mg HEA-NPs were irradiated under simulated sunlight of 1.0 kW m^–2^ (1 sun) and their surface temperatures were recorded through an infrared (IR) camera. As exhibited in Figs 3d and 4a, the surface temperature of the 21-HEA-NPs can increase to ∼107^o^C within 90 s and rapidly reduce to room temperature when the simulated light is removed. Such a phenomenon can also be recognized in other HEA-NPs under 1 sun irradiation, and it is found that the maximum surface temperatures of all the samples can exceed 100^o^C after ∼150 s irradiation (Fig. 4b), evidence for an excellent photothermal conversion performance.

### Water evaporation performance of HEA-NPs

**Figure 5.**
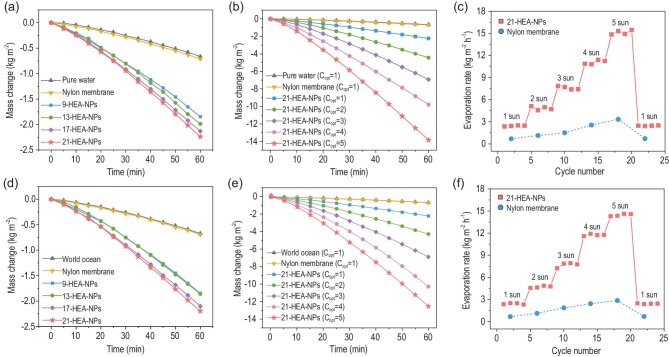
Photothermal conversion effect (water evaporation) of HEA-NPs. Water evaporation curves (pure water) for (a) different nanoparticles loaded on the nylon membrane under 1 sun irradiation, (b) 21-HEA-NPs loaded on the nylon membrane under 1–5 sun irradiation and (c) its corresponding stability, and the same performance in saline water (3.5 wt%), including (d) evaporation rates for different HEA-NPs under 1 sun, (e) 21-HEA-NPs under different irradiation and (f) its stability.

Owing to the great photothermal conversion performances, the water evaporation behavior of HEA-NPs was investigated by measuring the mass change of pure water under sunlight irradiation (Figs S20 and S21, Supplementary Data). To deeply understand the facilitation performance of HEA-NPs, the evaporation rates of pure water and original nylon membrane were calculated to be 0.67 and 0.69 kg m^–2^ h^–1^, respectively. Here, the evaporation rates of the 9-, 13-, 17- and 21-HEA-NPs under 1 solar irradiation (Fig. 5a) were analyzed to be 2.20, 2.26, 2.33 and 2.42 kg m^–2^ h^–1^, respectively, indicating that the evaporation rate of HEA-NPs can be improved through the ultra-mixing strategy of the composited elements. Moreover, the evaporation rate of 21-HEA-NPs has also been measured under the different simulated sunlight energy, in which the average water evaporation rate achieves 2.42, 4.84, 7.60, 11.10 and 15.12 kg m^–2^ h^–1^ under the light intensities of 1, 2, 3, 4 and 5 kW m^–2^, respectively (Fig. 5b and c, Fig. S22 and Table S4, Supplementary Data), indicating an excellent solar steam generation performance.

In addition, the evaporation rates of all the samples have been evaluated in saline water. The saline concentration was fixed at the average salinity of the world’s oceans (3.5 wt%). As shown in Fig. 5d, the evaporation rates of the 9-, 13-, 17- and 21-HEA-NPs under 1 solar irradiation in the saline water were calculated to be 2.17, 2.27, 2.29 and 2.43 kg m^–2^ h^–1^, respectively. Such a performance strongly suggests that the photothermal conversion performance of HEA-NPs cannot be influenced by inorganic salt. In addition, the evaporation rates of 21-HEA-NPs under different energy intensities (1 to 5 kW m^–2^) were calculated to be 2.43, 4.71, 7.70, 11.75 and 14.47 kg m^–2^ h^–1^, respectively (Fig. 5e and f, Fig. S22 and Table S4, Supplementary Data). It is noted that the HEA-NPs in saline water (3.5 wt%) have a similar photothermal conversion performance to those in pure water, indicating great potential for their application in seawater desalination.

**Figure 6.**
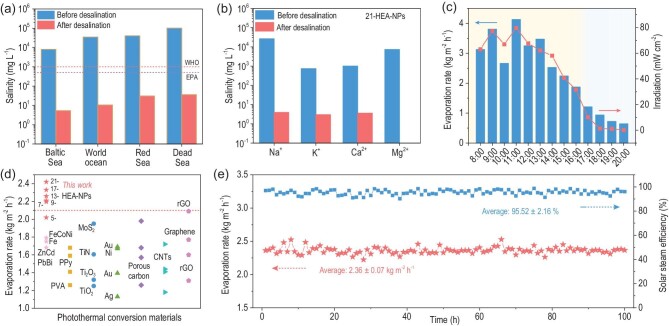
Desalination performance and outdoor application. (a) The measured salinities (the weight percentage of all ions) of the four simulated seawater samples before and after desalination. The dashed lines refer to the drinking water standards of the WHO and EPA. (b) The measured concentrations of four different ions (Na^+^, Mg^2+^, Ca^2+^, K^+^) of 21-HEA-NPs in saline water (3.5 wt%) before and after desalination. (c) Solar flux (dots) and freshwater yield per hour (bars) on a typical sunny day (28 August 2021, Shenyang). (d) Comparison of the photothermal evaporation rate of HEA-NPs and previously reported photothermal conversion materials. (e) Long-term stability test (100 h) of 21-HEA-NPs in 10 wt% saline water under 1 sun irradiation.

The solar steam efficiency of solar light (*η*), another important value for evaluating photothermal conversion performance, has been calculated based on Equation S3 (Supplementary Data) [7,33]. The average steam generation efficiency of the 9-, 13-, 17- and 21-HEA-NPs in pure water is 93.55%, 94.64%, 96.15% and 97.97%, respectively, and in saline water (3.5 wt%) is 93.17%, 93.25%, 96.20% and 97.83%, respectively (Table S5, Supplementary Data). Such a result indicates that efficiency can be improved along with the increase of composited elements. In particular, the steam generation efficiency of the 21-HEA-NPs could even exceed 99% during measurement, exhibiting an excellent photothermal conversion performance. To illustrate the excellent evaporation performance, the evaporation rates of HEA-NPs was compared to other previously reported materials (Table S6, ref. (S.M.) 30 to 53 in Supplementary Data), and the samples, including 7-, 9-, 13-, 17- and 21-HEA-NPs, possessed satisfactory water evaporation performance in the related reports (Fig. 6d).

The desalination performance of 21-HEA-NPs was evaluated in four artificial seawater samples with a salinity of 0.8 wt% (Baltic Sea), 3.5 wt% (world ocean), 4 wt% (Red Sea) and 10 wt% (Dead Sea) (Fig. S23, Supplementary Data) [7,34]. The average water evaporation rates of 21-HEA-NPs in 0.8, 3.5, 4 and 10 wt% seawater were 2.38, 2.46, 2.48 and 2.37 kg m^–2^ h^–1^, respectively (Fig. S23, Table S7, Supplementary Data), and the corresponding solar steam efficiencies were calculated to be 97.13%, 98.04%, 98.06% and 96.77% (Table S7, Supplementary Data). The concentration ions, including Na^+^, Mg^2+^, Ca^2+^ and K^+^, in collected water, have been reduced by about three orders of magnitude compared to that of simulated saline water before the desalination (Fig. 6a and b and Fig. S24, Supplementary Data). The salinity of collected water perfectly fits the standards of the World Health Organization (WHO) and the US Environmental Protection Agency (EPA) [35,36]. Therefore, the HEA-NPs are good candidates for photothermal conversion materials used for solar steam generating and seawater desalinating applications. Meanwhile, the long-term stability of the evaporation performance of 21-HEA-NPs has been evaluated in 10 wt% saline water under 1 sun irradiation. It was recognized that the evaporation rate and solar steam efficiency were stabilized at ∼2.36 kg m^–2^ h^–1^ and 95.52% after 100 h continuous measurement, respectively (Fig. 6e and Fig. S25, Supplementary Data), indicating that the HEA-NPs could serve as excellent desalination materials.

The photothermal conversion performance of the HEA-NPs was enhanced after the ultra-mixing of composited elements. Such an enhancement can be attributed to two main factors, including the *d–d* IBTs and the lattice distortion effect (high-entropy induced). Commonly, the energy of *d* bands in transition metals is gradually moved below the Fermi level when electron is increasing [17,37]. For example, the 3*d* bands of Fe, Co and Ni are located around the Fermi level, Ti, V and Cr are above the Fermi level, and Mn and Cu are below the Fermi level. Therefore, through composed with the 3*d* elements from different energy regions in the 21-HEA-NPs, a stronger *d–d* IBT can be realized, in which the energy of *d* bands around the Fermi level can be fully filled [38,39], resulting in an improved solar energy absorption property [17]. In our previous report, we showed that the energy regions were fully filled in 7-HEA-NPs (FeCoNiTiVCrCu), which demonstrated an excellent photothermal conversion (water evaporation rate) of 2.26 kg m^–2^ h^–1^ under 1 sun irradiation [20]. It is known that the addition of main group elements can reduce the performance of IBTs [31,32], however, the performance of 21-HEA-NPs was increased to 2.43 kg m^–2^ h^–1^. Thus, the IBT is not the only factor dominating photothermal conversion performance; it can be optimized via the lattice distortion effect. In general, there are four types of high-entropy effect—the high-entropy, the lattice distortion, the sluggish diffusion and the ‘cocktail’ effect [13,14,40–42]—which result in excellent performances characterized by, for example, good ductility, corrosion resistance and high strength of the HEAs [25,43,44]. Here, the lattice distortion effect of HEAs could result in the random occupation of metallic atoms in a crystalline structure, reducing the electrical and thermal conductivity [42,45,46]. The reduction of thermal conductivity can result in the accumulation of heat under solar irradiation, increasing the surface temperature of nanoparticles and thus enhancing the water evaporation rate. As demonstrated in XRD results (Fig. 1b), it can be concluded that lattice distortion has occurred with the addition of larger-atomic-radius elements including Ta, W, Pb and Bi. Consequently, the thermal conductivity of all the samples was carried out (Table S8, Supplementary Data), and it can be noted that the values were reduced with the increase of composited elements. In particular, the thermal conductivity of 21-HEA-NPs was reduced by >15% compared to that of 7-HEA-NPs, showing that the enhancement of photothermal conversion performance is associated with the coupling between IBTs and lattice distortion effect.

### Outdoor application of 21-HEA-NPs

Finally, to visually demonstrate the water evaporation performance of 21-HEA-NPs in the natural environment, the experimental equipment was placed on the terrace of the gymnasium at Shenyang (41.76^o^ N, 123.42^o^ E) for outdoor tests on a typical sunny day (28 August 2021) (Fig. S26, Supplementary Data). The temperature on that day was 17–28^o^C with an III-level southwesterly wind. The incident sunlight flux, evaporation rate, temperature and mass changing were recorded and are exhibited in Fig. 6c and Fig. S27 (Supplementary Data). The surface temperature of the HEA-NPs rose to the maximum value of 48.8^o^C at noon while the evaporation rate reached 4.13 kg m^–2^ h^–1^ under ∼0.80 sun irradiation. On a cloudy day, in comparison, the evaporation behavior was reduced, but still remained at a 2.24 kg m^–2^ h^–1^ rate under 0.57 sun irradiation (Fig. S27, Supplementary Data). In addition, the 21-HEA-NPs enable an evaporation rate of 0.72 and 0.50 kg m^–2^ h^–1^ at night on a sunny and cloudy day, respectively. It can be concluded that 21-HEA-NPs exhibit excellent photothermal conversion performance in the natural environment, and can be used in other photic applications.

## CONCLUSION

In summary, a series of HEA-NPs with 9, 13, 17 and 21 composited elements have been synthesized through an arc-discharged plasma method with the assistance of the *V.P.* strategy. In particular, a record ultra-mixed 21-element HEA-NP was successfully synthesized from a strongly repelling system, which exhibited a BCC structure and uniformly elemental distribution. Meanwhile, due to the *d–d* IBTs and the lattice distortion effect, such HEA-NPs demonstrated excellent photothermal conversion performance; the surface temperature reached 107^o^C within 90 s under simulated sunlight irradiation (1 sun). The evaporation rate and photothermal conversion efficiency are 2.42 kg m^–2^ h^–1^ and 99%, respectively, under 1 solar irradiation, demonstrating the highest photothermal conversion performance among all the HEA-NPs. Our work provides new insights into HEA-NP discovery and optimization, introducing a new approach to manipulating the compositions of high-entropy alloy and thus achieving differentiated performance design. Meanwhile, the relationship between photothermal conversion performance and high-entropy effect has been clarified, and could be expanded to other solar-based applications.

## MATERIALS AND METHODS

### Arc-discharged plasma method for synthesizing HEA-NPs

The arc-discharged method was achieved by an ultra-fast-cooling rate arc-discharged furnace designed by our research group [22,23]. The original metallic powders (purity in Table S9) were first pressed into the cylinder shape and then placed into the vacuum chamber. The reaction gas H_2_ and Ar were introduced into the chamber after the vacuum degree was reduced to 5 × 10^–3^ Pa. The arc-discharge process would be sustained for ∼5–10 min to make sure that the metallic powders had been reproduced into nanoparticles. The cooling collection plate was set at 300 and 150 K by employing water and liquid nitrogen as the cooling media. The as-made nanoparticles were collected after passivating for ∼6 h and then stored in an ambient environment.

### Structural characterizations

The microstructure and morphology of the nanoparticles were characterized by field-emission transmission electron microscope (FE-TEM, JEOL JEM-2100F, JEOL ARM-200 and Thermo Scientific Talos F200S) with an accelerating voltage of 200 kV. As for the preparation of the TEM sample, the nanoparticles were firstly dispersed in the ethanol and then dropped on the copper mesh. The particle size was measured by ImageJ software. XRD was performed using a Smart Lab9kW (Rigaku) with a scan step of 0.04^o^ in the 2*θ* region of 20^o^ to 90^o^. XPS was performed on a Thermo ESCALAB 250. The metal contents of HEA-NPs were analyzed by ICP-OES (PerkinElmer Optima 5300DV). The solutions were prepared by digesting the samples in a mixture of hydrochloric acid (HCl), hydrofluoric acid (HF) and perchloric acid (HClO_4_). The thermal conductivity was tested through a thermal constant analyzer (TPS 2500S, Hot Disk) at 40^o^C.

### Photothermal conversion performance tests

Solar absorption performance was characterized by ultraviolet-visible spectroscopy (UV-3600, Shimadzu) with an attached integrating sphere (ISR-3100) in the UV-vis-NIR region. The absorption spectra were obtained by measuring the diffuse reflectance recorded (*A*% = 1–*R*%). IR thermal images were collected by the HIKMICROH36, and the solar conversion performance was measured via an IR thermometer (SA-D2580A, Wuxi Shiao Technology Co., Ltd.). The water evaporation rate was measured based on the mass loss using an electronic mass balance (FA3204C, 0.1-mg accuracy) with a communication model RS322 connected with the computer. A Xe light with AM 1.5 filter (1.0 to 5.0 kW m^–2^, CEL-PF300L-3A, Beijing Education Au-light Co., Ltd.) was employed as the simulated sunlight. The light intensity was monitored by a full-spectrum optical power meter (CEL-FZ-A, Beijing Education Au-light Co., Ltd.). The surface temperature of HEA-NPs was recorded by the SA-D2580A and a recorder (SA-JLY01A4, Wuxi Shiao Technology Co., Ltd.) for 60 min with an interval of 1 s. The simulated seawater, with salinity equaling the average of the world’s oceans, was prepared by dissolving 1.314 g NaCl, 0.037 g KCl, 0.05 g CaCl_2_, 0.305 g MgCl_2_·6H_2_O and 0.059 g MgSO_4_ in 50 mL deionized water. Other simulated seawaters (Baltic Sea, Red Sea, Dead Sea) were prepared by scaling up and down the salinity of the simulated world's oceans.

## Supplementary Material

nwac041_Supplemental_File

## References

[1] Alvarez PJ , Chan CK, Elimelech M et al. Emerging opportunities for nanotechnology to enhance water security. Nat Nanotechnol 2018; 13: 634–41. 10.1038/s41565-018-0203-2 30082804

[2] Elimelech M , Phillip WA. The future of seawater desalination: energy, technology, and the environment. Science 2011; 333: 712–7. 10.1126/science.1200488 21817042

[3] Jassby D , Cath TY, Buisson H. The role of nanotechnology in industrial water treatment. Nat Nanotechnol 2018; 13: 670–2. 10.1038/s41565-018-0234-8 30082807

[4] Li J , Wang X, Lin Z et al. Over 10 kg m^−2^ h^−1^ evaporation rate enabled by a 3D interconnected porous carbon foam. Joule 2020; 4: 928–37. 10.1016/j.joule.2020.02.014

[5] Zhou L , Li X, Ni GW et al. The revival of thermal utilization from the sun: interfacial solar vapor generation. Natl Sci Rev 2019; 6: 562–78. 10.1093/nsr/nwz030 34691905 PMC8291486

[6] Zhou L , Tan Y, Ji D et al. Self-assembly of highly efficient, broadband plasmonic absorbers for solar steam generation. Sci Adv 2016; 2: e1501227. 10.1126/sciadv.1501227 27152335 PMC4846456

[7] Zhou L , Tan Y, Wang J et al. 3D self-assembly of aluminium nanoparticles for plasmon-enhanced solar desalination. Nat Photonics 2016; 10: 393–8. 10.1038/nphoton.2016.75

[8] Mi B . Interfacial solar evaporator for brine treatment: the importance of resilience to high salinity. Natl Sci Rev 2021; 8: nwab118. 10.1093/nsr/nwab118 34858615 PMC8566170

[9] Politano A , Argurio P, Di Profio G et al. Photothermal membrane distillation for seawater desalination. Adv Mater 2017; 29: 1603504. 10.1002/adma.201603504 28066987

[10] Hessel CM , Pattani VP, Rasch M et al. Copper selenide nanocrystals for photothermal therapy. Nano Lett 2011; 11: 2560–6. 10.1021/nl201400z 21553924 PMC3111000

[11] Gao M , Zhu L, Peh CK et al. Solar absorber material and system designs for photothermal water vaporization towards clean water and energy production. Energy Environ Sci 2019; 12: 841–64. 10.1039/C8EE01146J

[12] Xie P , Yao Y, Huang Z et al. Highly efficient decomposition of ammonia using high-entropy alloy catalysts. Nat Commun 2019; 10: 4011. 10.1038/s41467-019-11848-9 31488814 PMC6728353

[13] Ye YF , Wang Q, Lu J et al. High-entropy alloy: challenges and prospects. Mater Today 2016; 19: 349–62. 10.1016/j.mattod.2015.11.026

[14] George EP , Raabe D, Ritchie RO. High-entropy alloys. Nat Rev Mater 2019; 4: 515–34. 10.1038/s41578-019-0121-4

[15] Li Z , Zhai L, Ge Y et al. Wet-chemical synthesis of two-dimensional metal nanomaterials for electrocatalysis. Natl Sci Rev 2021; doi: 10.1093/nsr/nwab142. 10.1093/nsr/nwab142 PMC9113131 35591920

[16] Zhong G , Xu S, Dong Q et al. Rapid, universal surface engineering of carbon materials via microwave-induced carbothermal shock. Adv Funct Mater 2021; 31: 2010968. 10.1002/adfm.202010968

[17] Li Y , Liao Y, Zhang J et al. High-entropy-alloy nanoparticles with enhanced interband transitions for efficient photothermal conversion. Angew Chem Int Ed 2021; 60: 27113–8. 10.1002/anie.202112520 34605601

[18] Frey NA , Peng S, Cheng K et al. Magnetic nanoparticles: synthesis, functionalization, and applications in bioimaging and magnetic energy storage. Chem Soc Rev 2009; 38: 2532–42. 10.1039/b815548h 19690734 PMC2740941

[19] Li Z , Pradeep KG, Deng Y et al. Metastable high-entropy dual-phase alloys overcome the strength–ductility trade-off. Nature 2016; 534: 227–30. 10.1038/nature17981 27279217

[20] Yao Y , Huang Z, Hughes LA et al. Extreme mixing in nanoscale transition metal alloys. Matter 2021; 4: 2340–53. 10.1016/j.matt.2021.04.014

[21] Yao Y , Huang Z, Xie P et al. Carbothermal shock synthesis of high-entropy-alloy nanoparticles. Science 2018; 359: 1489–94. 10.1126/science.aan5412 29599236

[22] Li Y , Chen X, Wei Q et al. Oxygen-sulfur co-substitutional Fe@c nanocapsules for improving microwave absorption properties. Sci Bull 2020; 65: 623–30. 10.1016/j.scib.2020.01.009 36659131

[23] Li Y , Liu R, Pang X et al. Fe@C nanocapsules with substitutional sulfur heteroatoms in graphitic shells for improving microwave absorption at gigahertz frequencies. Carbon 2018; 126: 372–81. 10.1016/j.carbon.2017.10.040

[24] Guo S , Liu CT. Phase stability in high entropy alloys: formation of solid-solution phase or amorphous phase. Prog Nat Sci Mater Int 2011; 21: 433–46. 10.1016/S1002-0071(12)60080-X

[25] Lee C , Chou Y, Kim G et al. Lattice-distortion-enhanced yield strength in a refractory high-entropy alloy. Adv Mater 2020; 32: 2004029. 10.1002/adma.202004029 33135322

[26] Song H , Tian F, Hu QM et al. Local lattice distortion in high-entropy alloys. Phys Rev Mater 2017; 1: 023404. 10.1103/PhysRevMaterials.1.023404

[27] Alcock C , Itkin V, Horrigan M. Vapour pressure equations for the metallic elements: 298–2500k. Can Metall Q 1984; 23: 309–13. 10.1179/cmq.1984.23.3.309

[28] Ahn M , Park Y, Lee SH et al. Memristors based on (Zr, Hf, Nb, Ta, Mo, W) high-entropy oxides. Adv Electron Mater 2021; 7: 2001258. 10.1002/aelm.202001258

[29] Xu X , Guo Y, Bloom BP et al. Elemental core level shift in high entropy alloy nanoparticles via X-ray photoelectron spectroscopy analysis and first-principles calculation. ACS Nano 2020; 14: 17704–12. 10.1021/acsnano.0c09470 33284574

[30] Yao RQ , Zhou YT, Shi H et al. Nanoporous surface high-entropy alloys as highly efficient multisite electrocatalysts for nonacidic hydrogen evolution reaction. Adv Funct Mater 2021; 31: 2009613. 10.1002/adfm.202009613

[31] Legare MA , Belanger-Chabot G, Dewhurst RD et al. Nitrogen fixation and reduction at boron. Science 2018; 359: 896–9. 10.1126/science.aaq1684 29472479

[32] Liu S , Li Z, Wang C et al. Turning main-group element magnesium into a highly active electrocatalyst for oxygen reduction reaction. Nat Commun 2020; 11: 938. 10.1038/s41467-020-14565-w 32071314 PMC7028951

[33] Zhao L , Yang Q, Guo W et al. Co_2.67_S_4_-based photothermal membrane with high mechanical properties for efficient solar water evaporation and photothermal antibacterial applications. ACS Appl Mater Interfaces 2019; 11: 20820–7. 10.1021/acsami.9b04452 31117447

[34] Shao Y , Jiang Z, Zhang Y et al. All-poly(ionic liquid) membrane-derived porous carbon membranes: scalable synthesis and application for photothermal conversion in seawater desalination. ACS Nano 2018; 12: 11704–10. 10.1021/acsnano.8b07526 30398843

[35] Surwade SP , Smirnov SN, Vlassiouk IV et al. Water desalination using nanoporous single-layer graphene. Nat Nanotechnol 2015; 10: 459–64. 10.1038/nnano.2015.37 25799521

[36] World Health Organization . Safe Drinking-Water from Desalination. https://www.who.int/publications/i/item/WHO-HSE-WSH-11.03 (14 February 2022, date last accessed).

[37] Yuan Y , Dong C, Gu J et al. A scalable nickel-cellulose hybrid metamaterial with broadband light absorption for efficient solar distillation. Adv Mater 2020; 32: 1907975. 10.1002/adma.201907975 32159267

[38] Zhang L , Cai W, Bao N. Top-level design strategy to construct an advanced high-entropy Co-Cu-Fe-Mo(oxy)hydroxide electrocatalyst for the oxygen evolution reaction. Adv Mater 2021; 33: 2100745. 10.1002/adma.202100745 33876867

[39] Zhan C , Xu Y, Bu L et al. Subnanometer high-entropy alloy nanowires enable remarkable hydrogen oxidation catalysis. Nat Commun 2021; 12: 6261. 10.1038/s41467-021-26425-2 34716289 PMC8556242

[40] Batchelor TAA , Pedersen JK, Winther SH et al. High-entropy alloys as a discovery platform for electrocatalysis. Joule 2019; 3: 834–45. 10.1016/j.joule.2018.12.015

[41] Oses C , Toher C, Curtarolo S. High-entropy ceramics. Nat Rev Mater 2020; 5: 295–309. 10.1038/s41578-019-0170-8

[42] Xin Y , Li S, Qian Y et al. High-entropy alloys as a platform for catalysis: progress, challenges, and opportunities. ACS Catal 2020; 10: 11280–306. 10.1021/acscatal.0c03617

[43] Pang J , Zhang H, Zhang L et al. Ductile Ti_1.5_ZrNbAl_0.3_ refractory high entropy alloy with high specific strength. Mater Lett 2021; 290: 129428. 10.1016/j.matlet.2021.129428

[44] Qiu Y , Thomas S, Fabijanic D et al. Microstructural evolution, electrochemical and corrosion properties of Al_x_CoCrFeNiTi_y_ high entropy alloys. Mater Des 2019; 170: 107698. 10.1016/j.matdes.2019.107698

[45] Kim G , Diao H, Lee C et al. First-principles and machine learning predictions of elasticity in severely lattice-distorted high-entropy alloys with experimental validation. Acta Mater 2019; 181: 124–38. 10.1016/j.actamat.2019.09.026

[46] Nutor RK , Cao Q, Wang X et al. Phase selection, lattice distortions, and mechanical properties in high-entropy alloys. Adv Eng Mater 2020; 22: 2000466. 10.1002/adem.202000466

