# Supplementary material for: High-entropy-alloy nanoparticles with 21 ultra-mixed elements for efficient photothermal conversion
Source: Natl Sci Rev. 2022 Mar 4;9(6):nwac041. doi: 10.1093/nsr/nwac041 (PMC9170356; doi:10.1093/nsr/nwac041)
Supplement: nwac041_Supplemental_File [file nwac041_supplemental_file.docx]

*Supporting Information for*

**High-entropy-alloy nanoparticles with ultra-mixed 21 elements for efficient photothermal conversion**

***Yijun Liao ^1,#^, Yixing Li ^1,#,^*, Rongzhi Zhao ^1,2^, Jian Zhang ^2^, Lizhong Zhao ^2^, Lianze Ji ^1,2^, Zhengyu Zhang ^1^,*** ***Xiaolian Liu ^2^, Gaowu Qin ^1^, Xuefeng Zhang ^1,2,^****

***^#^*** *These authors* *contributed equally to this work.*

*^1^ Key Laboratory for Anisotropy and Texture of Materials (MOE), School of Materials Science and Engineering, Northeastern University, Shenyang 110819, P. R. China.*

*^2^ Institute of Advanced Magnetic Materials, College of Materials and Environmental Engineering, Hangzhou Dianzi University, Hangzhou 310012, P. R. China.*

*^*^Corresponding author.* [*liyx@mail.neu.edu.cn*](mailto:liyx@mail.neu.edu.cn) *(Y. Li), zhang@hdu.edu.cn (X. Zhang)*

**Supplementary Text**

*Configuration entropy of mixing*

The configurational entropy of mixing for multi-compositional nanoparticles can be expressed by the following equation (Eq. S1) (1, 2):

${\Delta S}_{mix}=-R\sum_{i=1}^{n} X_{i}lnX_{i}$ (S1)

Where Δ*S*_mix_ is the configurational entropy of mixing, *R* is the gas constant, *X_i_* is the molar ratio of component *i*, and *n* is the total number of elements involved. As shown in Fig. 2F, the mixing entropy shows an approximately linear relationship. With the increase of the alloying element amount, the mixing entropy (Δ*S*_mix_) also increases linearly: the FeCoNi alloys stand in medium entropy (1R<Δ*S*_mix_<1.5R), while others stand in high entropy classifications (Δ*S*_mix_>1.5R) (3). Based on the arc-discharged method, the configurational entropy of 21-HEA-NPs could exceed 24.55 J/mol/K, which is 2.69 times that of FeCoNi nanoparticles (9.12 J/mol/K).

*XRD analysis*

The crystalline structural uniformity of different HEA-NPs has been confirmed by XRD analysis (Fig. 1C and Fig. S3). Because the HEA-NPs are composited by the strongly immiscible elements, there are no XRD standard cards in literature or in the XRD library to reference/compare.

For different HEA-NPs, the XRD peaks exhibit a slight difference with increasing the composited element number. Three obvious peaks in the XRD pattern of 21-HEA-NPs were observed at around 42.5^o^, 61.8^o^, and 76.8^o^, respectively, which is obeyed the crystal structure rule of BCC structure (4). It can be recognized that the crystalline structure for the HEA-NPs with less than 9 composited elements is FCC structure, while that transform into BCC structure when the composited elements exceed 9.

*Microstructures of HEA-NPs*

The particle sizes of different HEA-NPs have been counted by the *ImageJ* software based on the low-resolution TEM images (Figs. S5), as shown in Fig. S6. The diameters of 9-, 13-, 17-, and 21- HEA-NPs are centered at 75, 72, 82, 87, and 62 nm, respectively, which can be seen that the average diameter of HEA-NPs is centered at ~80 nm. All nanoparticles are in a sphere-like shape, and it can be concluded that the particle size could be reduced via the ultra-fast cooling treatment.

*Vapor pressure of the composited metals*

As for the bulk metal, the theoretical vapor pressure versus temperature in the range of 1000 to 3000 K (Fig. S1) could be calculated by Eq. 2 (5, 6):

$\log\left( \frac{\text{p}}{\text{atm}} \right)\text{=}\text{A}\text{+}\frac{\text{B}}{\text{T}}\text{+}\text{C}\text{×}\log\left( \text{T} \right)\text{+}\frac{\text{D}}{\text{T}^{\text{3}}}$ (S2)

where *p* is the vapor pressure, *atm* is the atmospheric pressure, *T* is the temperature, and *A*, *B*, *C*, and *D* are the fitting parameters.

*High temperature (T) strategy for synthesizing HEA-NPs*

To explain the strategy, the 3-element FeCoNi alloy nanoparticles (FeCoNi, denoted 3-HEA-NPs) were synthesized, and the elemental distribution was illustrated by the energy-dispersive X-ray spectroscopy (EDS) maps (Fig. S7). The FeCoNi-based nanoparticles with previously considered immiscible combinations (2), including Cr-Cu, Fe-Cu, V-Cu, were successfully prepared. A uniform mixing structure of FCC was confirmed by XRD and EDS maps (Fig.1C and Figs. S7-S8). The corresponding nanoparticles were marked as 5-HEA-NPs (FeCoNiCrCu) and 7-HEA-NPs (FeCoNiTiVCrCu), respectively. It is believed that the extreme-high synthesized temperature (high *T* strategy) could favor immiscible compositions to overcome the alloying rampart and thus enable the formation of HEA-NPs. To further verify the expanding ability, the 9-element HEA-NPs of FeCoNiCrYTiVCuAl with multi-repelling constitutions, such as Cr-Cu, Cr-Y, Y-Ti, Y-V, Fe-Cu, Cu-V (2), were synthesized. It has been proved that the uniform mixed nanoparticles were composed of all used metallic elements, but the crystalline structure was transformed into the body-centered-cubic (BCC) structure (Fig. 1C) (4).

*Vapor pressure (V.P.) strategy for the preparation HEA-NPs*

Based on the curves between vapor pressure and temperature, it can be recognized that the previously-reported 5-, 7- and 9-element HEANPs are all alloy from the elements in the *V.P._M_* group owing to the roughly identical evaporation rate (5), and it will change when the metals from *V.P._H_* and/or *V.P._L_* were introduced. To explore the contributions of vapor pressure more carefully, two control experiments were performed to prepare 4-element nanoparticles from *V.P._H_* or *V.P._L_* elements as well as 13-element nanoparticles from 9-*V.P._M_*-elements (9-HEA-NPs without *V.P.* design) and different *V.P._H_* or *V.P._L_* elements. Due to the formation of intermetallic compounds between Sn-Y and Sn-Cu would destroy the single-phase structure, Sn has been classified to the high vapor pressure group (Fig. S9).

Subsequently, the 4-element nanoparticles of NbMoTaW, CdZnBiPb, and InMnAgSn were prepared. The XRD and TEM-EDS maps (Fig. 2B and Figs. S2-S3) show that only NbMoTaW nanoparticles exhibit a uniform mixed structure, while the others have separated phases. As for 13-element nanoparticles, the uniform mixed structure was only observed in the product that combined with NbMoTaW but the Nb and Mo maintain a low concentration, as demonstrated by the EDS mapping (Figs. S3 and S10). And for the rest nanoparticles combined with ZnCdPbBi or AgInMnSn, Zn, Cd, and/or Mn clusters were formed inside the nanoparticles, resulting in the phase-separated behavior (Figs. S11 and S12).

Typically, the element with high vapor pressure could achieve a high evaporated rate during the synthesized process and thus result in the enrichment on the collected plates/formation sites (3, 7, 8). Such phenomenon could be further reinforced under the high *T* synthesized strategy, in which the metals would be preferentially evaporated under extreme-high temperature and thus result in the formation of clusters within nanoparticles (3). To solve such holdback, the optimization on vapor pressure takes an important position and it meanwhile has been reported that the total vapor pressure of combinations could be tailored via the composited elements (9). Therefore, the adjustment on the raw element ratios of different *V.P.* metals could optimize the evaporated behavior during the preparation process (*V.P.* design), such as the reduced *V.P._H_* contents and simultaneously increased *V.P._L_* contents, which might enable the formation of uniformly mixed among the above repelling combinations (10, 11).

To verify the availability of the *V.P.* design, the amounts of different metals were adjusted in the following synthesis. Three 13-element nanoparticles including FeCoNiCrYTiVCuAlNbMoTaW, FeCoNiCrYTiVCuAlZnCdPbBi and FeCoNiCrYTiVCuAlAgInMnSn were obtained under the arc-discharged process. The corresponding raw powder ratio of NbMoTaW, ZnCdPbBi and AgInMnSn was fixed at 10, 0.1 and 0.5 respectively (marked as FeCoNiCrYTi_10_V_10_Cu_0.5_Al_0.5_ + Nb_10_Mo_10_Ta_10_W_10_ / Zn_0.1_Cd_0.1_Pb_0.1_Bi_0.1_ / Ag_0.5_In_0.5_Mn_0.5_Sn_0.5_). It was found that only the products synthesized from the *V.P._L_* metals (NbMoTaW) demonstrate a uniform mixed structure (Fig. 2D and Fig. S3 and S13-S14) with significantly increased contents of Nb and Mo. As for other nanoparticles, the crystalline structure synthesized from *V.P._H_* metals exhibits the uniformly composited tendency as compared to those nanoparticles prepared without *V.P.* design. These results indicate that the evaporation rate of ­*V.P._H_* metals could not be significantly reduced by adjusting the raw ratio and adding *V.P._M_* metals, which should be modified via a further reduced vapor pressure. Therefore, the *V.P._L_* elements as the key materials were used to further optimize the evaporation rate for alloying the immiscible combinations together. Surprisingly, the 17-element nanoparticles were successfully prepared by combining 13-HEA-NPs (NbMoTaW) with either ZnCdPbBi or AgInMnSn, and products exhibit a uniform mixed structure (BCC) with uniform distribution of composited elements in each nanoparticle (Fig. 2E and Fig. S4). It can be concluded that the additional *V.P._L_* metals can optimize the evaporated process of the mixed precursor under the plasma irradiation, and which is distinct from the previously-reported alloying methods based on the mixing enthalpy design (3, 12).

*Control experiments I: 4-element nanoparticles*

We hypothesized that the vapor pressure of the compositional elements is the fundamental reason for triggering the separated phase in the nanoparticles. To verify this, control experiments were conducted using the metallic elements in the different *V.P.* groups (5). The metals of the *V.P._H_* group have been divided into two groups according to the difference of intrinsic vapor pressure (CdZnBiPb and InMnAgSn). Different nanoparticles were synthesized via the same prepared process of 9-HEA-NPs. Water was employed as the cooling medium.

According to the results of XRD and EDS maps (Figs. S2-S3 and Fig. 2B), it can be seen that the nanoparticles synthesized via *V.P._L_* metals exhibit an FCC structure and different elements exhibit a uniform distribution at the nanoscale. As for that of others, multiple phase structures can be observed in both nanoparticles while the FCC structure can be seen in AgInMnSn. In EDS maps, the Janus style can be seen in both nanoparticles, indicating that the evaporated rate of *V.P._H_* elements would result in the phase separation during the arc-discharged synthesis.

*Control experiments II: 13-element nanoparticles (without V.P. design)*

In addition to the 13-element nanoparticles without *V.P.* design, we prepared the corresponding nanoparticles to further confirm the necessity of the precise control of raw material contents. The nanoparticles have been prepared in the same packet mode of 4-element nanoparticles. The corresponding nanoparticles were synthesized under the same conditions with 9-HEA-NPs.

According to the results of XRD and EDS maps (Fig. S3 and S10-S12), the uniform mixed structure could only be seen in the nanoparticles with NbMoTaW, while the contents of Nb and Mo are relatively low. For other nanoparticles, a similar XRD pattern could be observed compared to Fig. S3. Some clusters such as Zn, Cd, and Mn appeared within the nanoparticle in the TEM-EDS maps, and which results in the macroscopical phase-separation. Therefore, it can be concluded that the HEA-NPs could not be synthesized without the *P.V.* design.

*Ultra-fast cooling strategy for synthesizing HEA-NPs*

Apart from the high *T* and *V.P.* design strategy, the fast-cooling rate is another key factor in creating the non-equilibrium process for synthesizing HEA-NPs (12, 13). The composited metals can be uniformly evaporated and mixed under the high synthesized temperature while the subsequent cooling process could maintain the homogeneous structure of the prepared nanoparticles (14, 15). However, the phase-separation occurs in the 21-element HEA-NPs obtained by the high *T* and *V.P.* design strategy equipping with the conventional cooling process (Figs. S15 and S16). Commonly, the local structural rearrangement during the cooling process could promote the formation of a uniform mixed structure, while the long-range solute partition would react in the phase separation (3, 12). Therefore, the corresponding phenomenon is related to the relatively long synthesized time, which could deteriorate the cooling rate during the formation process (Fig. 1A), meanwhile leading to a new challenge for the cooling system. Faced with this challenge, liquid nitrogen as the cooling medium was used to activate the thermodynamic quenching (12).

*Control experiments III: 21-element nanoparticles*

To further confirm the effectiveness of the combinations of high *T*, *V.P.* design, and ultra-fast cooling strategy, the 21-element nanoparticles without *P.V.* design (denoted 21-E-NPs without *P.V.* design) and with *P.V.* design and conventional cooling (denoted 21-E-NPs conventional cooling) were prepared by the arc-discharged plasma method under the same conditions with 9-HEA-NPs.

For the 21-E-NPs without the *P.V.* design, chaos phase structures were appeared in the XRD pattern (Fig. S3). Several elements were enriched in the nanoparticles as shown in EDS maps (Fig. S15). The corresponding result reveals that the immiscible metallic system cannot form the uniform alloy without the *P.V.* design. As for 21-E-NPs conventional cooling, the XRD pattern has been significantly optimized after the *P.V.* design, which is similar to that of 13-element nanoparticles (AgInMnSn). In the EDS maps (Fig. S16), the uniform distribution of most elements indicated that the chaos distribution of composited elements has been rearranged by the *P.V.* design. Therefore, it can be concluded that the *V.P.* design is the most important process to realize the alloy forming in the strongly immiscible metallic system.

*The element ratio of HEA-NPs*

To characterize the uniformity of elements in 21-HEA-NPs, the STEM EDS maps (using Cu grid) have been detected in three different regions, in which the elemental ratios have been listed as follow (at%):

Region 1: Fe (1.42%), Co (1.45%), Ni (0.92%), Cr (0.89%), Y (0.43%), Ti (12.97%), V (16.78%), Cu (*51.26%*), Al (0.47%), Nb (2.04%), Mo (5.7%), Ta (1.19%), W (1.14%), Zn (0.56%), Cd (0.28%), Pb (0.33%), Bi (0.27%), Ag (0.34%), In (0.53%), Mn (0.55%), Sn (0.48%);

Region 2: Fe (2.73%), Co (2.35%), Ni (1.28%), Cr (1.30%), Y (3.69%), Ti (13.36%), V (11.8%), Cu (*56.68%*), Al (3.48%), Nb (0.44%), Mo (0.74%), Ta (0.12%), W (0.24%), Zn (0.18%), Cd (0.17%), Pb (0.15%), Bi (0.18%), Ag (0.10%), In (0.16%), Mn (0.65%), Sn (0.20%);

Region 3: Fe (3.42%), Co (2.64%), Ni (3.22%), Cr (2.33%), Y (2.74%), Ti (16.20%), V (14.82%), Cu (*47.53%*), Al (0.88%), Nb (1.14%), Mo (1.49%), Ta (0.48%), W (0.67%), Zn (0.58%), Cd (0.09%), Pb (0.60%), Bi (0.12%), Ag (0.12%), In (0.55%), Mn (0.26%), Sn (0.12%).

A slightly different elemental distribution could be observed, which indicates that the composited elements for each nanoparticle are not absolutely the same, ascribing to the synthesized method is a free diffusion process.

*XPS analysis*

The XPS analysis was performed on the 21-HEA-NPs to illustrate the surface state of different elements (Figs. S17 to S19). According to different fitting peaks, both metal bonds and oxidized bonds have been discovered in different HEA-NPs, evidencing that the surface of nanoparticles has been oxidized after being stored in an ambient environment (16-20).

Note that the detective depth of XPS is about 5 nm, which could only reveal the surface elemental distributions of the HEA-NPs. For the elemental composition ratios, we will not specifically use XPS to verify the ratio but the STEM elemental maps and/or ICP-OES results.

*Photothermal conversion performance*

The solar steam efficiency of solar light (*η*) can be calculated by the following equation (21, 22):

$\eta=\frac{\dot{m}(C\Delta T+\Delta h)}{q_{i}}$ (S3)

where *ṁ* is the solar-driven evaporation rate of water under solar illumination (*ṁ*= *m_light_*- *m_dark_*), *q_i_* is the incident power density of solar illumination, *C* is the specific heat capacity of water (4.18 J g^-1^ K^-1^), Δ*T* is the increased temperature of the water, and Δ*h* is the enthalpy of vaporization at the relative temperature.

For the water evaporated experiment, the maximum temperature was reduced to around 44.0 ^o^C. The surface temperature could reach the maximum temperature in 60 min under 1.0 kW m^-2^ simulated solar light (Fig. S20). As for the water evaporated performance, the evaporation rate of pure water was set as the control group and the value under 1.0 kW m^-2^ has been measured to be 0.51 kg m^-2^ h^-1^. As for the nylon substrate, the evaporation rate of that is 0.65 kg m^-2^ h^-1^ (Fig. S21). The surrounding temperature was set at 25 ^o^C. Under different simulated solar energy (1 to 5 kW m^-2^), the surface temperatures for 21-HEA-NPs could reach 44.2, 55.6, 63.8, 67.5, and 70.9 ^o^C, respectively (Fig. S22). Furthermore, the same characterization of evaporation rate with different simulated solar energy (1 to 5 kW m^-2^) has also been carried out in the average salinity of the world ocean (3.5 wt%). The surface temperatures reach 44.0, 57.6, 66.6, 68.5, and 71.5 ^o^C, respectively (Fig. S20).

In seawater desalination performance, the weight percentage of Na^+^ ions has decreased to 2.73, 3.92, 18.16, and 17.19 mg L^-1^ of four different simulated seawater, respectively (Fig. S24). As for that of K^+^ and Ca^2+^ ions, the weight percentage was reduced to 2.63, 2.98, 3.7, 5.75 mg L^-1^ for K^+^ and 0, 3.58, 8.63, 11.93 mg L^-1^ for Ca^2+^, respectively. Meanwhile, only 1.19 mg L^-1^ Mg^2+^ ions were detected in the Dead Sea samples. It can be noted that the evaporation rate of HEA-NPs has been slightly reduced in the saline water, but the HEA-NPs could still satisfy the requirement of the seawater desalinated materials.

**Supplementary Figures**


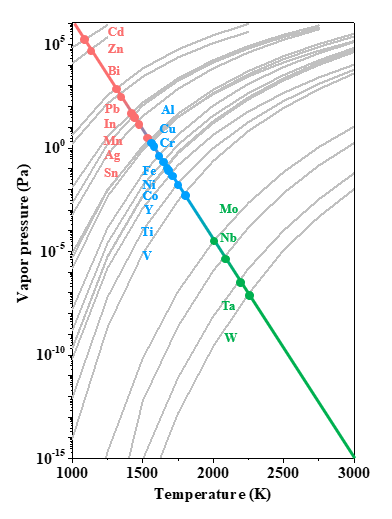


**Fig. S1.** Vapor pressure versus temperature of different metallic elements in this work, in which the elements have been classified into high vapor pressure, medium vapor pressure, and low vapor pressure group.


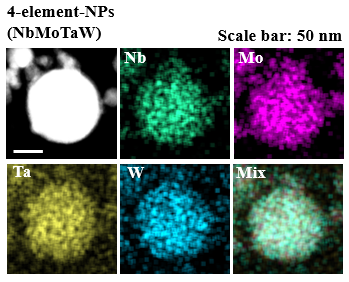


**Fig. S2.** STEM and the corresponding EDS elemental maps of NbMoTaW nanoparticles.


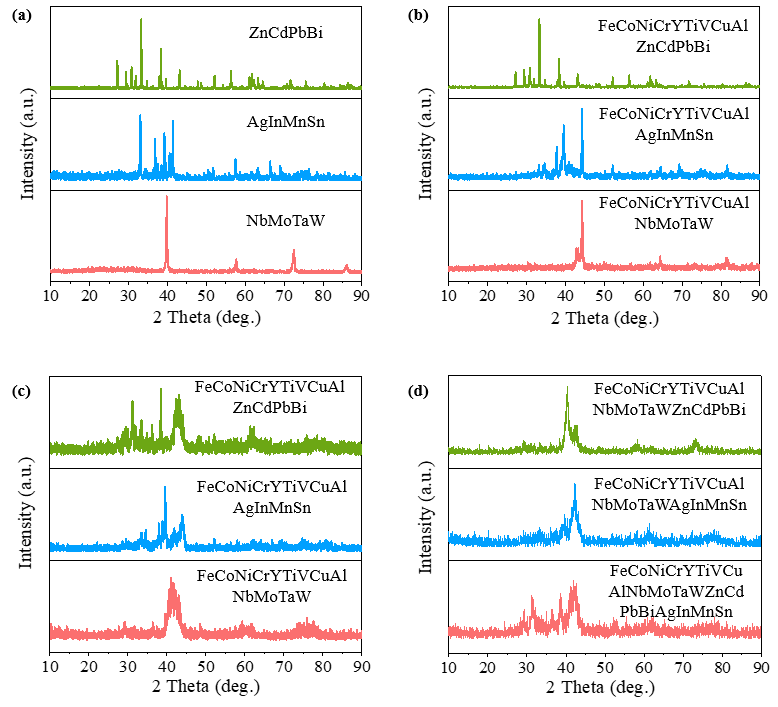


**Fig. S3.** XRD patterns of the different nanoparticles. (a) 4-element. (b) 13-element without V.P. strategy. (c) 13-element with V.P. strategy. (d) 17-element and 21-element (conventional cooling).


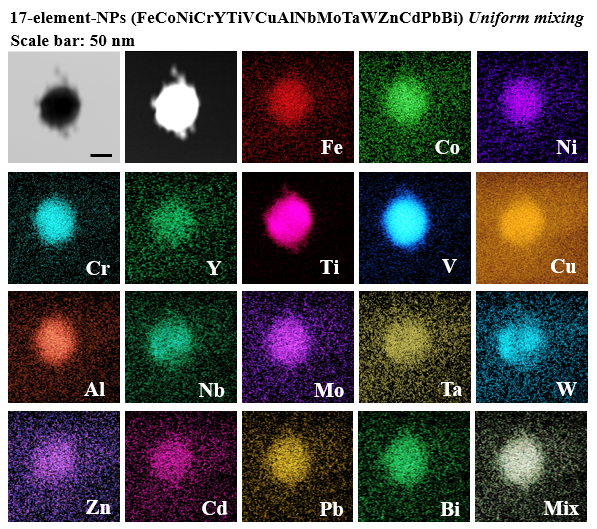


**Fig. S4.** STEM and the corresponding EDS elemental maps of 17-element nanoparticles (FeCoNiCrYTiVCuAlNbMoTaWZnCdPbBi) with *V.P.* design and high-entropy driven.

**
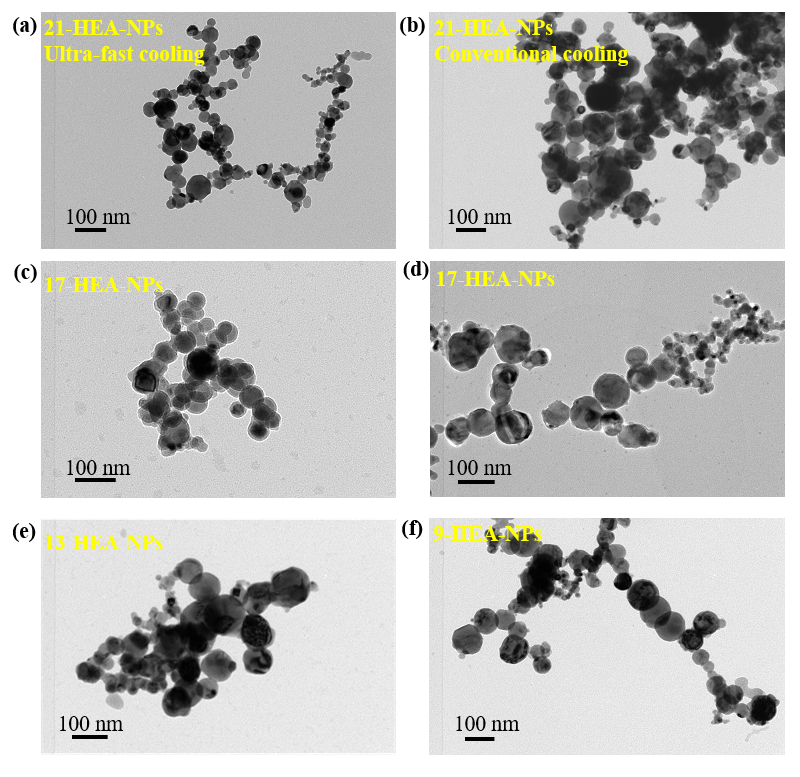
**

**Fig. S5.** Low-resolution TEM images of different HEA-NPs.

**
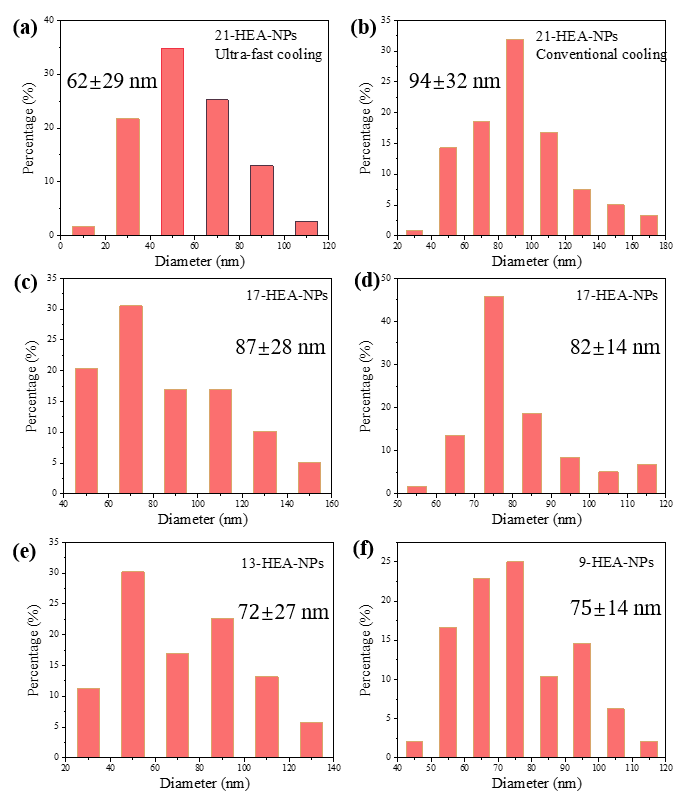
**

**Fig. S6.** The corresponding statistic results of TEM images of different HEA-NPs.


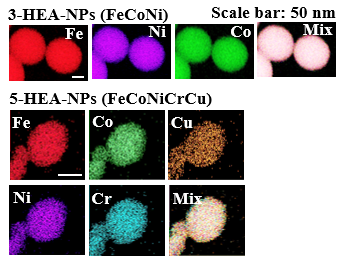


**Fig. S7.** STEM and the corresponding EDS elemental maps of 3-element (FeCoNi) and 5-element (FeCoNiCrCu) nanoparticles.


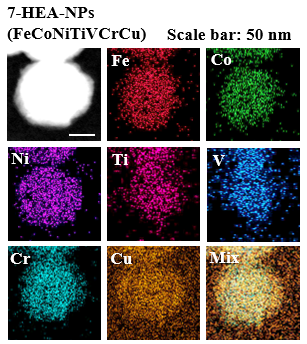


**Fig. S8.** STEM and the corresponding EDS elemental maps of 7-element (FeCoNiTiVCrCu) nanoparticles.


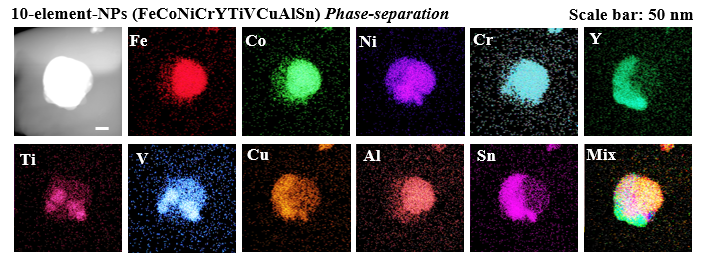


**Fig. S9.** STEM and the corresponding EDS elemental maps of the 10-element-NPs (FeCoNiCrYTiVCuAlSn), in which the phase separation raised by Sn, Y, and Cu could be observed.


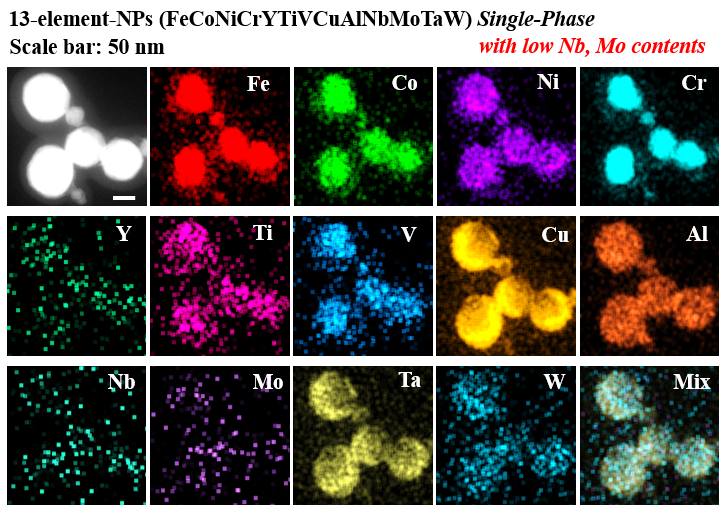


**Fig. S10.** STEM and the corresponding EDS elemental maps of the 13-element nanoparticles (FeCoNiCrYTiVCuAlNbMoTaW), in which only a slight content of Nb and Mo could be detected.


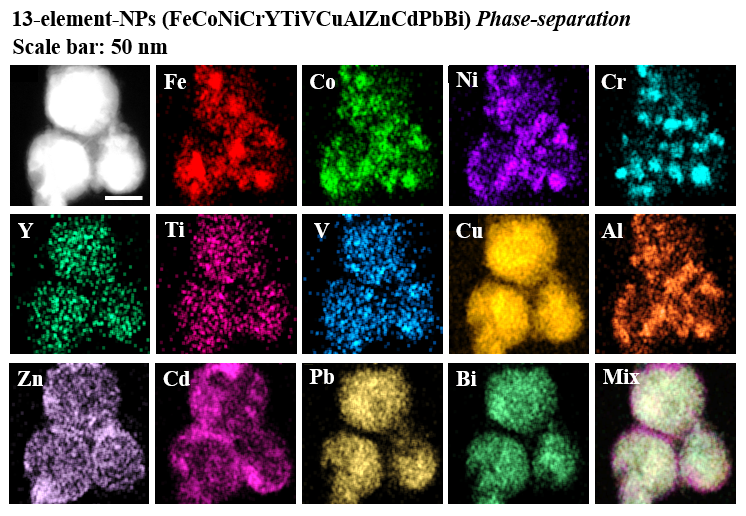


**Fig. S11.** STEM and the corresponding EDS elemental maps of the 13-element nanoparticles (FeCoNiCrYTiVCuAlZnCdPbBi).


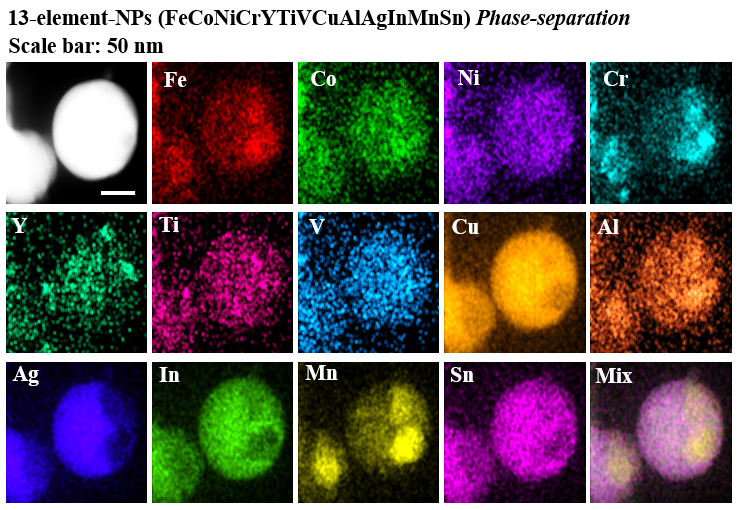


**Fig. S12.** STEM and the corresponding EDS elemental maps of the 13-element nanoparticles (FeCoNiCrYTiVCuAlAgInMnSn).

**
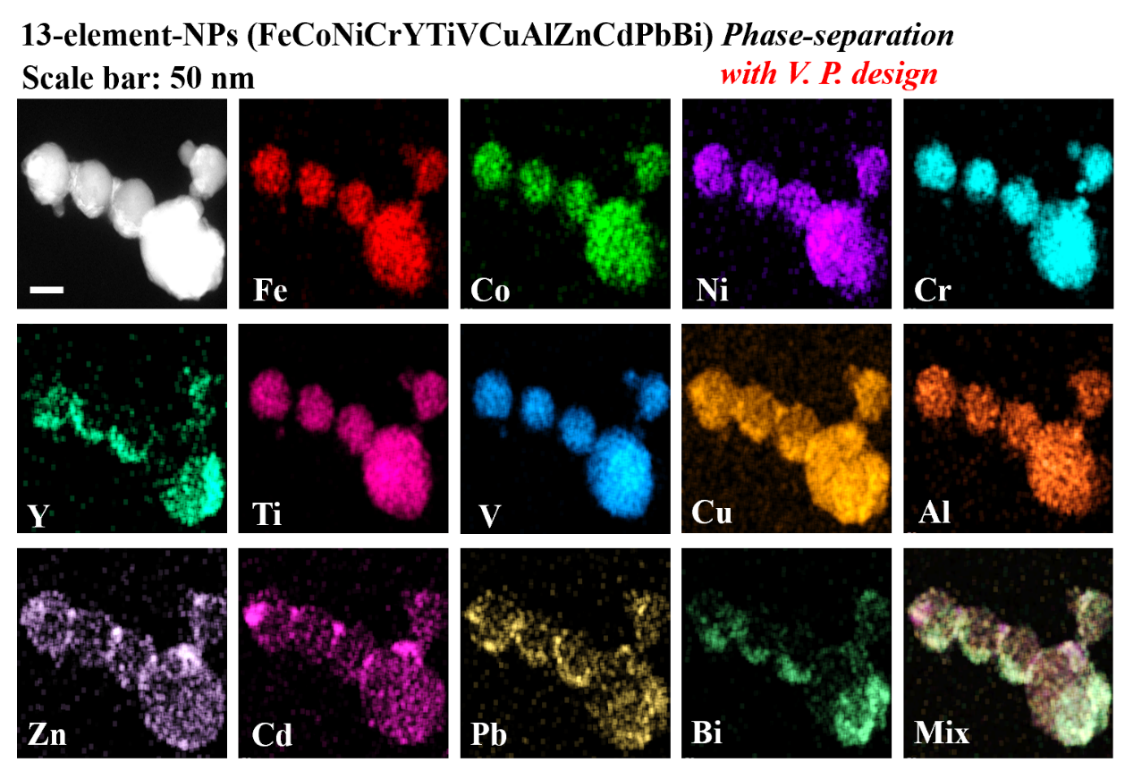
**

**Fig. S13.** STEM and the corresponding EDS elemental maps of the 13-element nanoparticles (FeCoNiCrYTiVCuAlZnCdPbBi) with *V.P.* design.

**
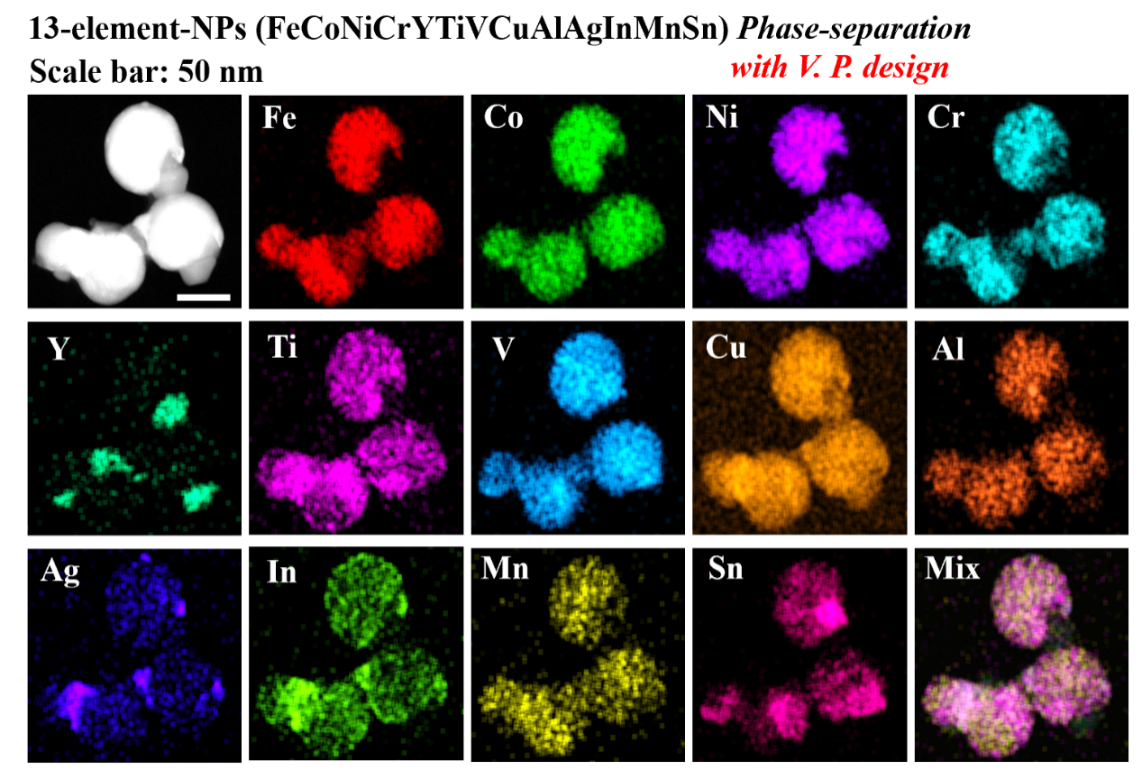
**

**Fig. S14.** STEM and the corresponding EDS elemental maps of the 13-element nanoparticles (FeCoNiCrYTiVCuAlAgInMnSn) with *V.P.* design.

**
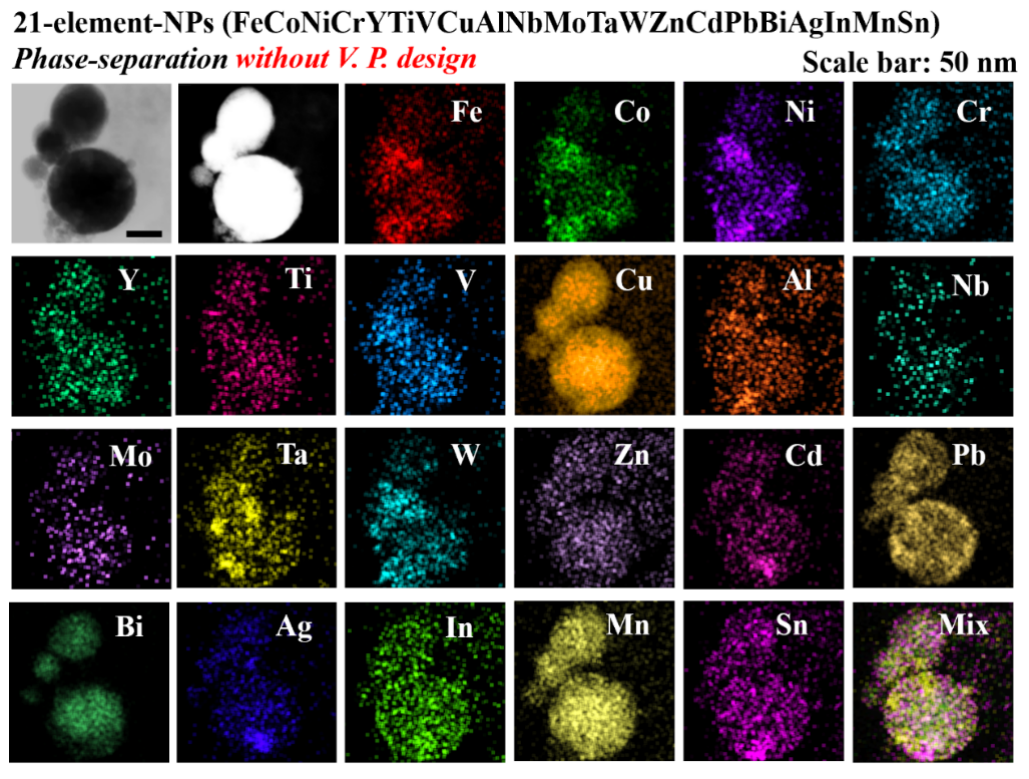
**

**Fig. S15.** STEM DF images and the corresponding EDS elemental maps of 21-element nanoparticles (FeCoNiCrYTiVCuAlNbMoTaWZnCdPbBiAgInMnSn) without *V.P.* design.

**
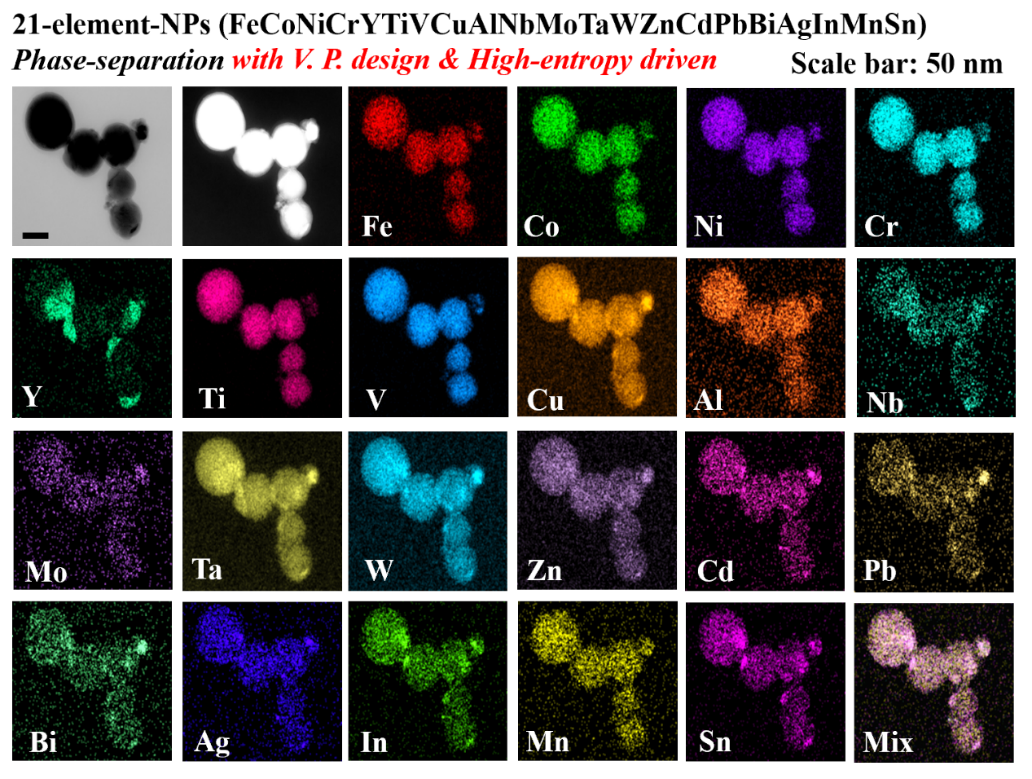
**

**Fig. S16.** STEM DF images and the corresponding EDS elemental maps of 21-element nanoparticles (FeCoNiCrYTiVCuAlNbMoTaWZnCdPbBiAgInMnSn) with *V.P.* design and high-entropy driven.


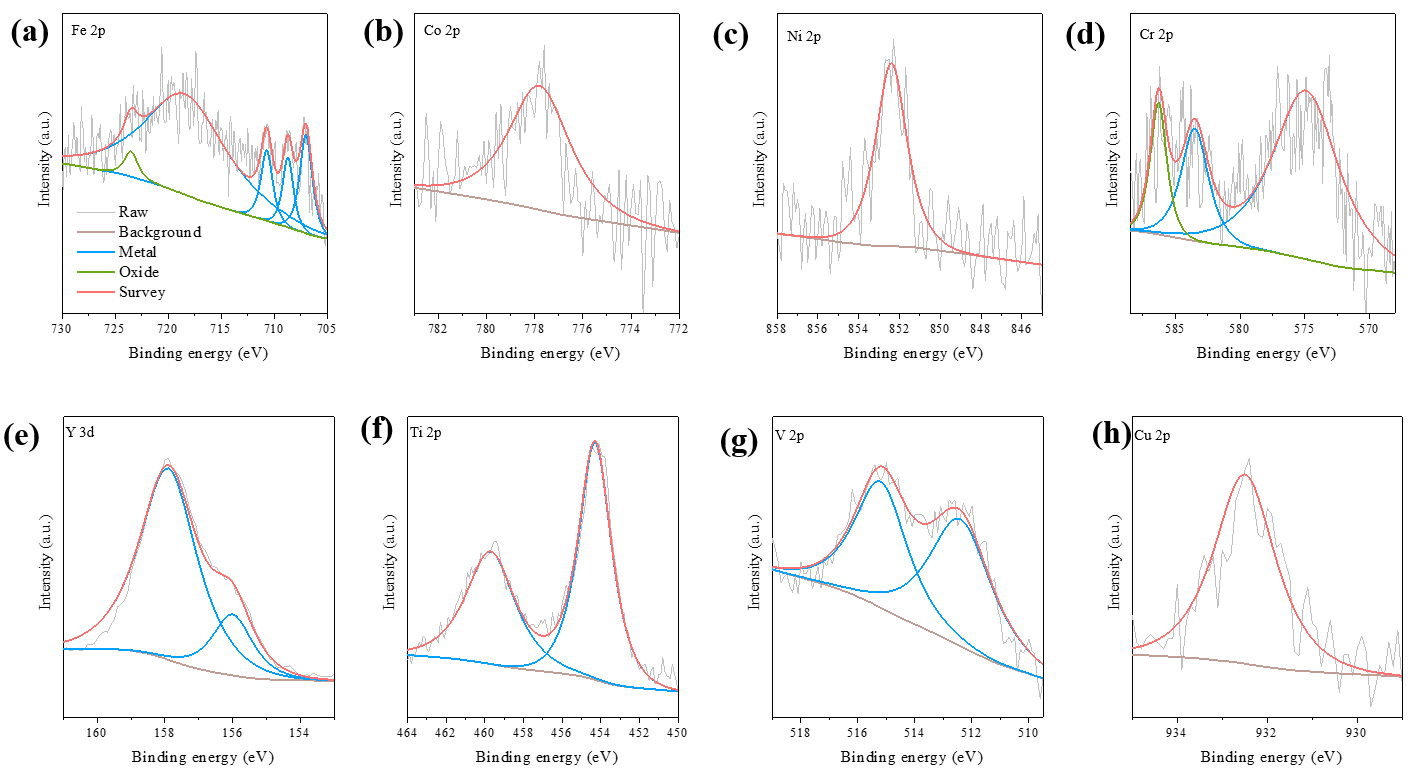


**Fig. S17.** Characteristic XPS peaks and the corresponding summarized table for Fe, Co, Ni, Cr, Y, Ti, V, and Cu from the 21-HEA-NPs.


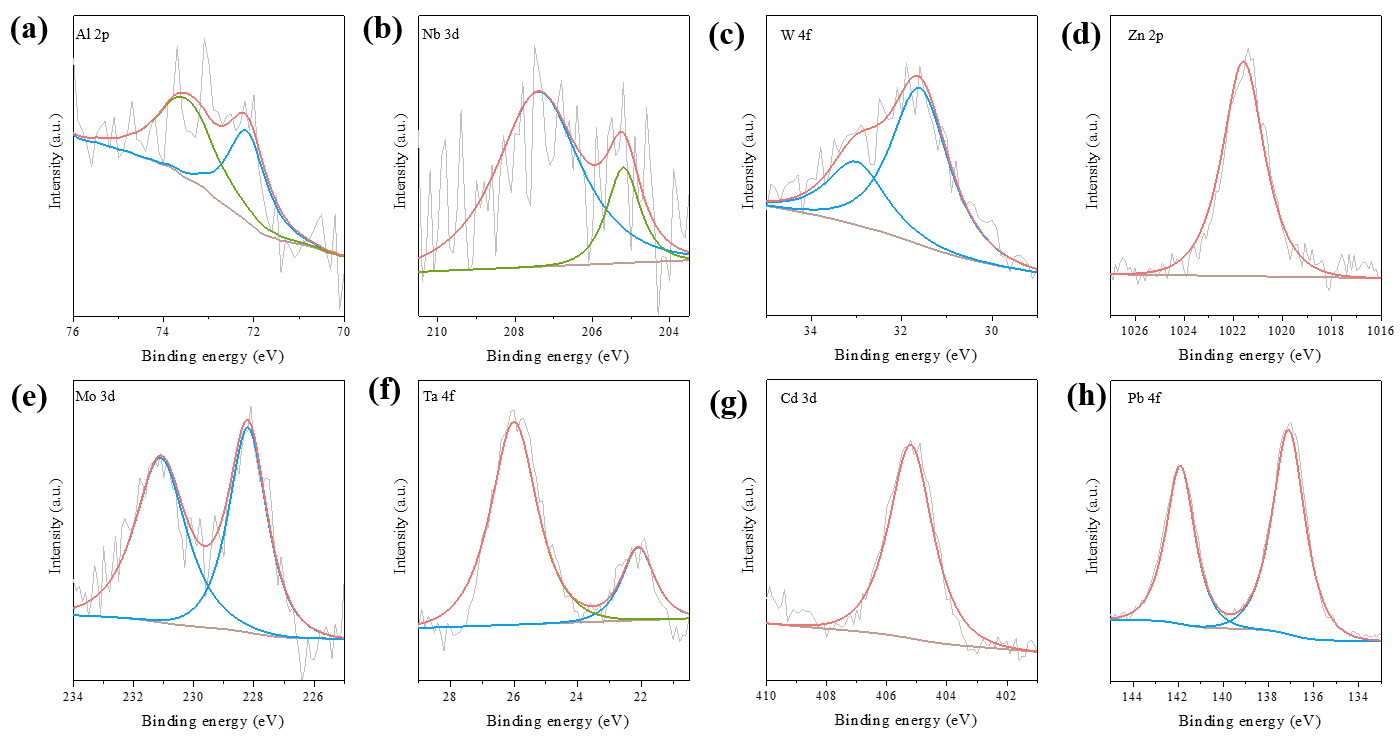


**Fig. S18.** Characteristic XPS peaks and the corresponding summarized table for Al, Nb, Mo, Ta, W, Zn, Cd, and Pb from the 21-HEA-NPs.


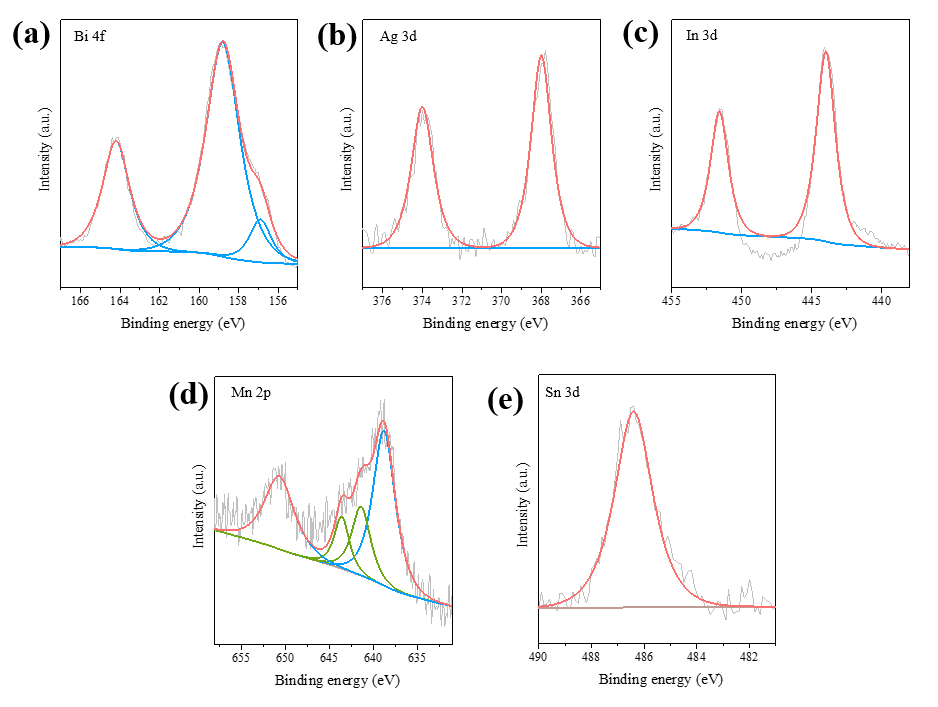


**Fig. S19.** Characteristic XPS peaks and the corresponding summarized table for Bi, Ag, In, Mn, and Sn from the 21-HEA-NPs.


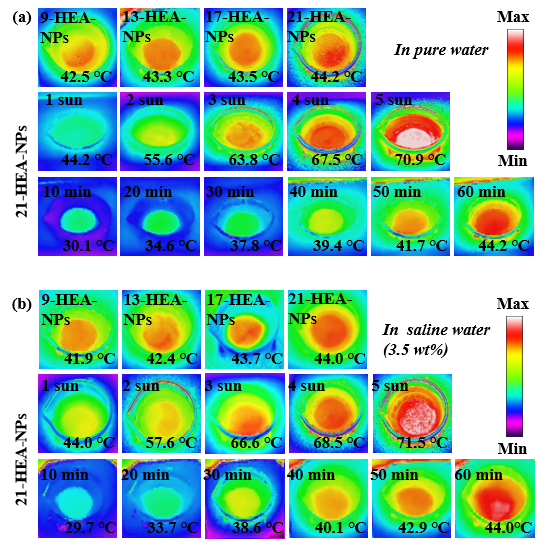


**Fig. S20.** Steady-state thermal images of the HEA-NPs on the pure (a) and saline (b) water, carried out by an IR camera, in which the temperature is the maximum heating site.


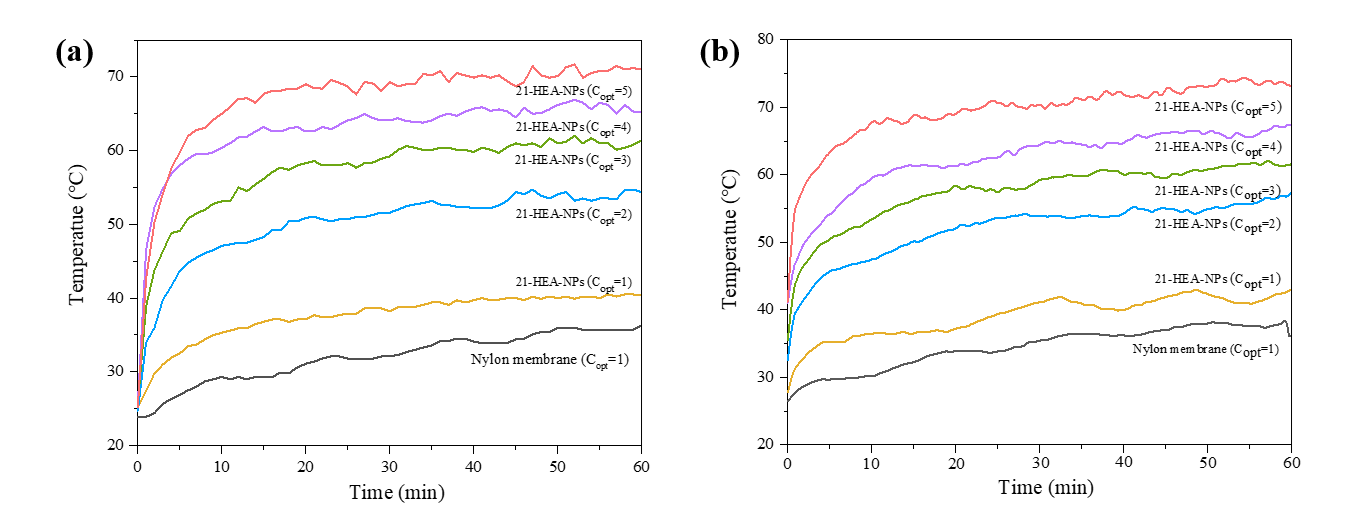


**Fig. S21.** The temperature change tendency of 21-HEA-NPs under different solar irradiation in (a) pure and (b) saline water.

**
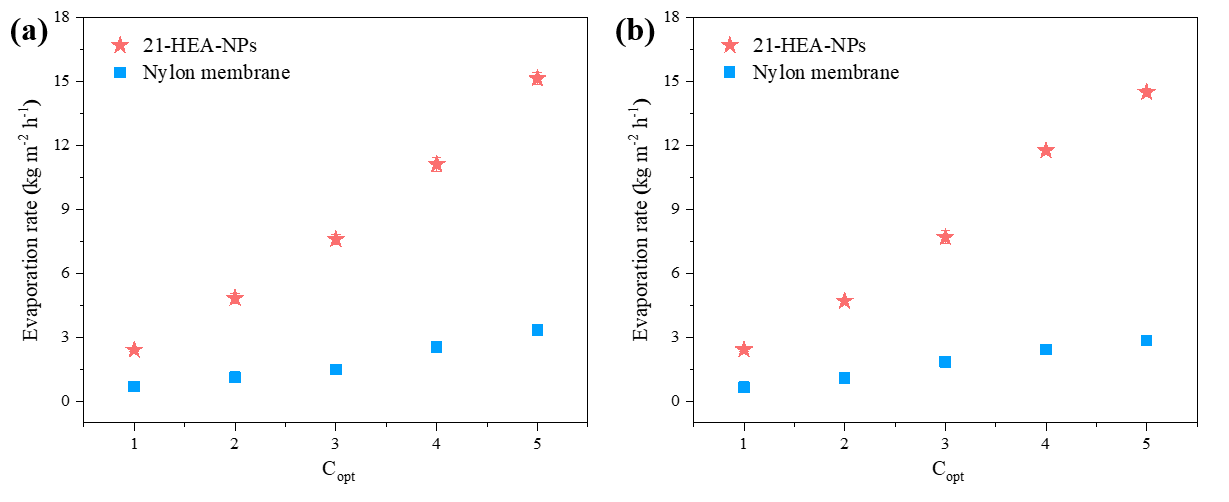
**

**Fig. S22.** Water evaporation curves for 21-HEA-NPs under different solar concentrations (1 to 5 sun) in pure (a) and saline water (3.5 wt%) (b).


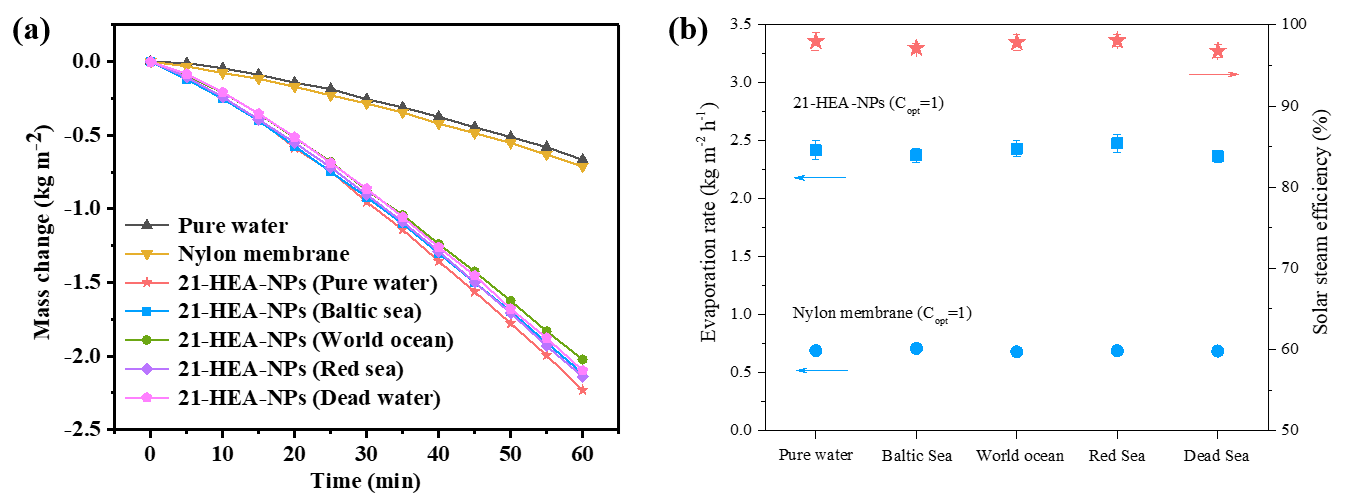


**Fig. S23.** (a) Mass change tendency, (b) evaporation rate, and solar steam efficiency of 21-HEA-NPs under 1 sun in different salinities.


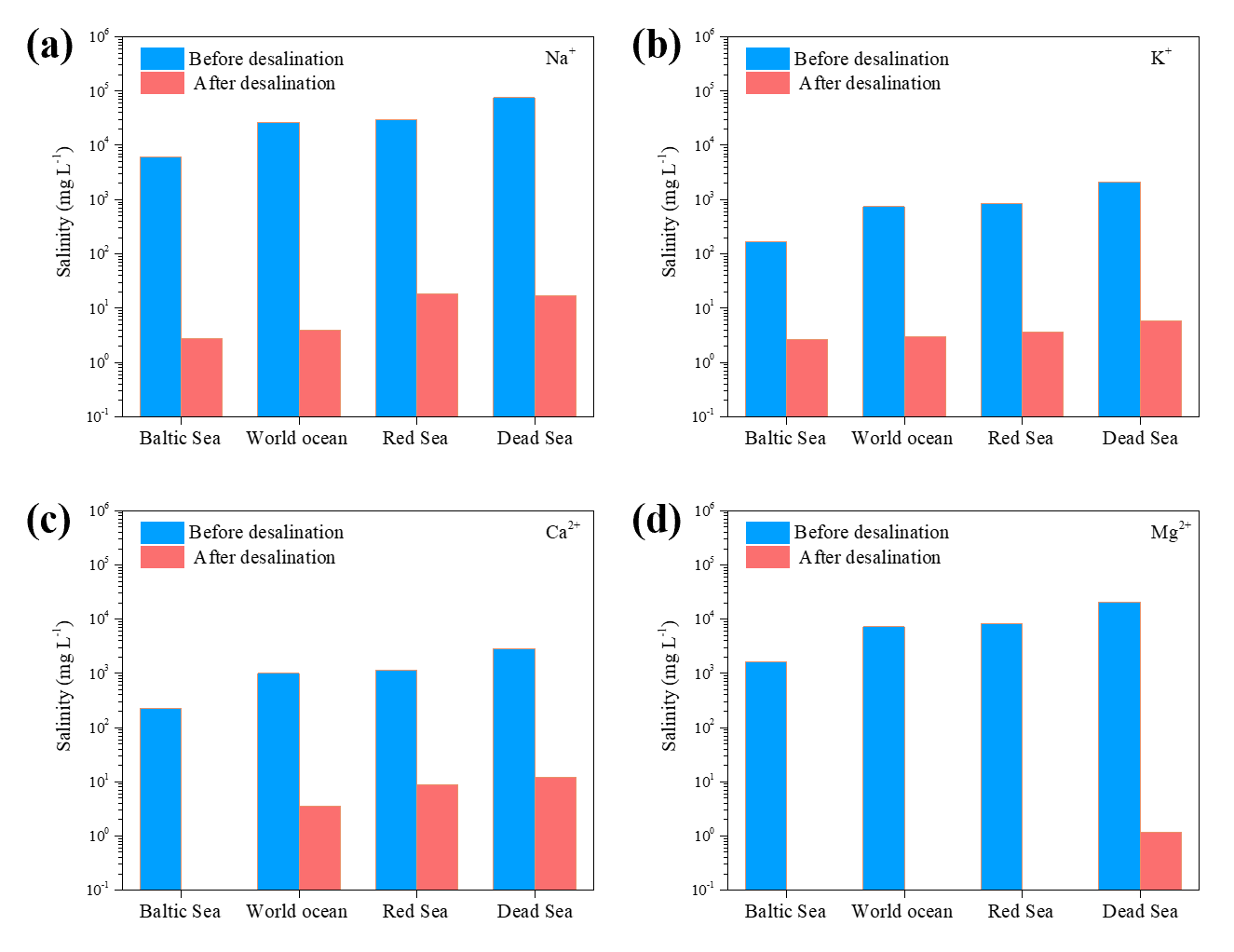


**Fig. S24.** The measured concentrations of four different ions (Na^+^, Mg^2+^, Ca^2+^, K^+^) in four different simulated salinities before and after desalination.


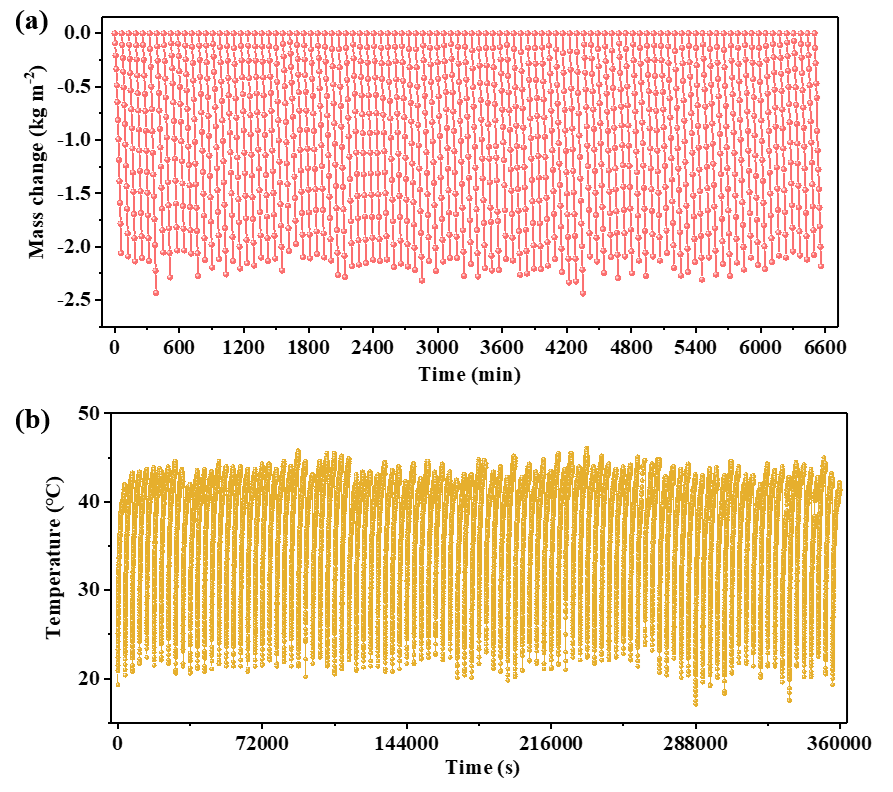


**Fig. S25.** Long-term stability test (100 h) of 21-HEA-NPs in 10 wt% saline water under one sun irradiation. (a) Mass change of water. (b) The corresponding changing of surface temperature.


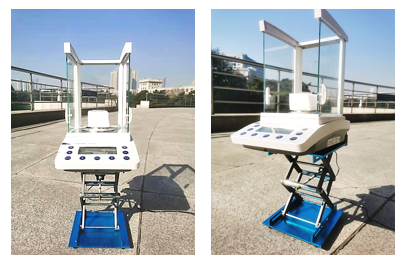


**Fig. S26.** Digital photograph of the outdoor evaporation performance test.


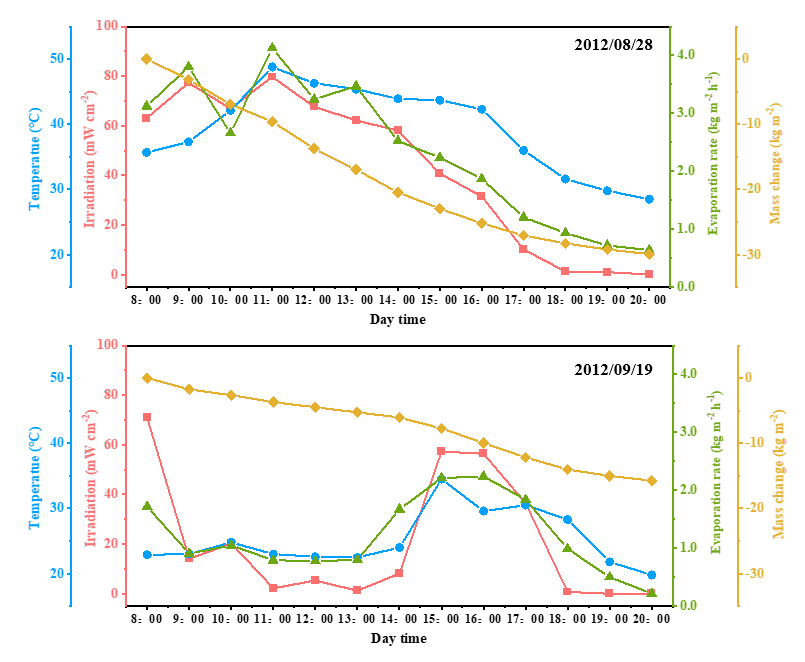


**Fig. S27.** Outdoor conditions for evaporation: incident sunlight flux, temperature, mass changing, and evaporation rate in a typical sunny (a) and cloudy (b) day.

**Table S1.** Composited elemental contents for different HEA-NPs measured by ICP-OES.


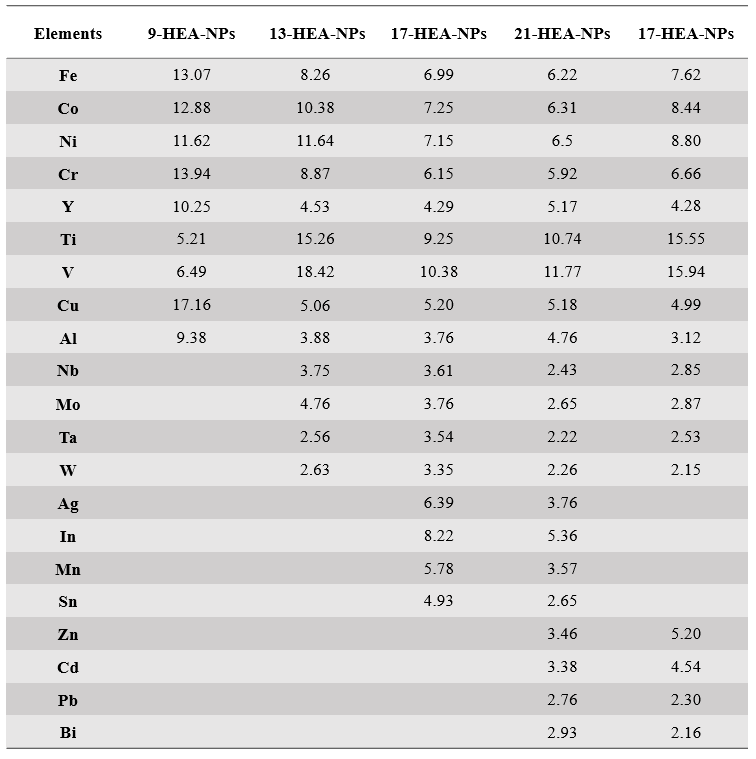


**Table S2.** Configurational entropy (Δ*S*_mix_) of 9-, 13-, 17-, and 21-HEA-NPs.

**
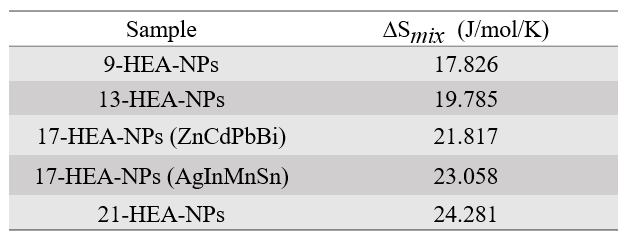
**

**Table S3.** Summarization of optical absorption performances of HEA-NPs and carbon-based materials (23-29).


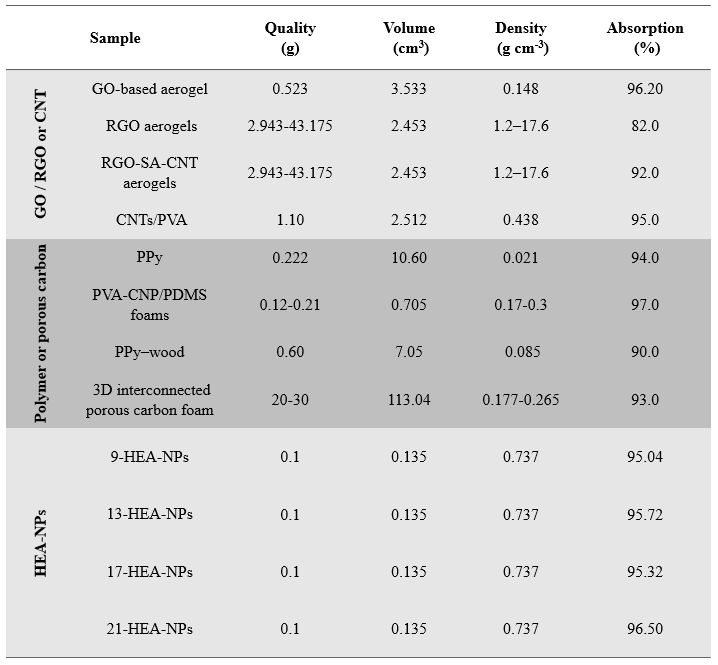


**Table S4.** Summarization of photothermal conversion performances of HEA-NPs (pure and saline water).


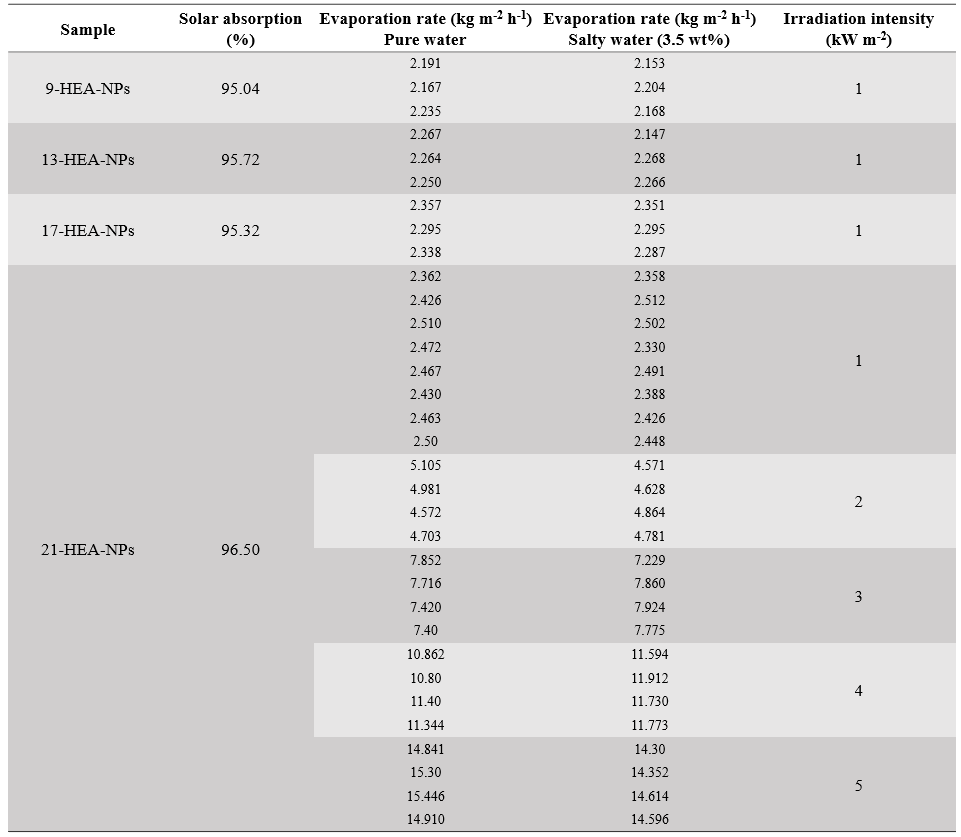


**Table S5.** Summarization of solar steam efficiency of HEA-NPs (pure and saline water).


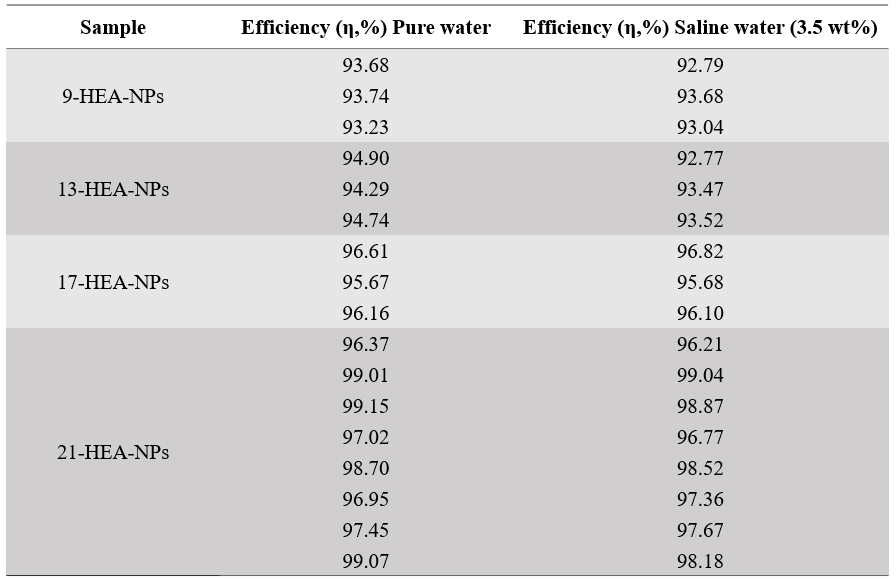


**Table S6.** Solar-driven water evaporation performances of different materials under one sun (30-53).


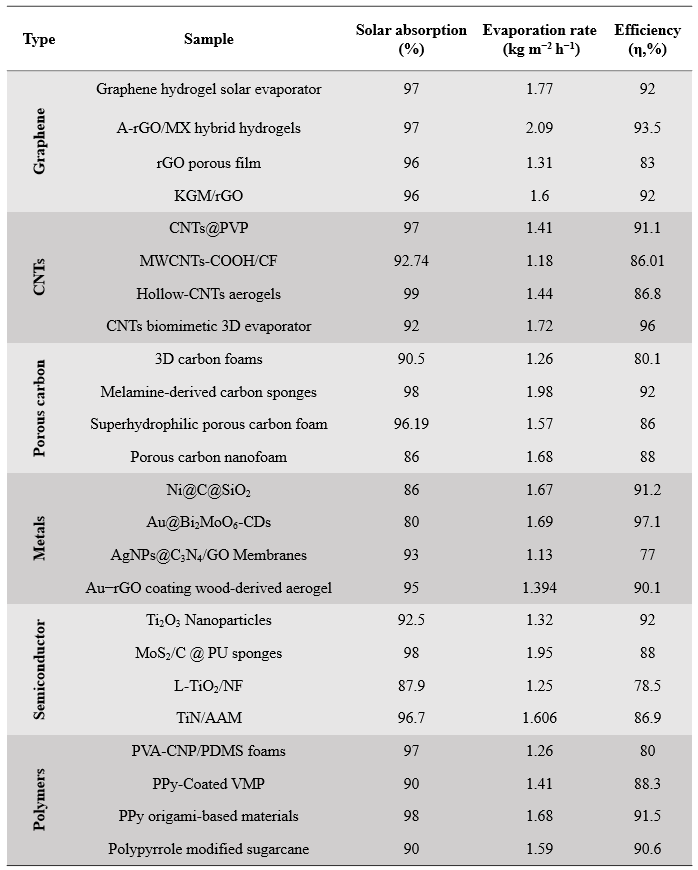


**Table S7.** Summarization of photothermal conversion performances of HEA-NPs in other saline water (0.8 wt%, 4 wt%, and 10 wt%).


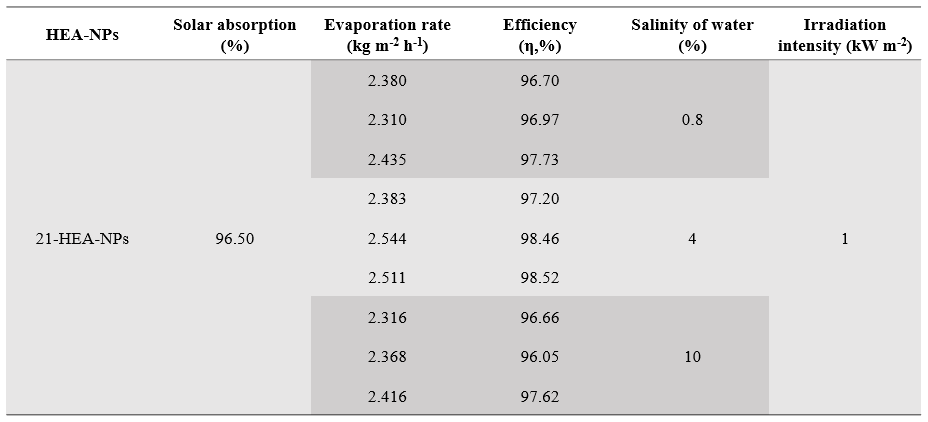


**Table S8.** Thermal conductivity (λ) of 7-, 9-, 13-, 17-, and 21-HEA-NPs.


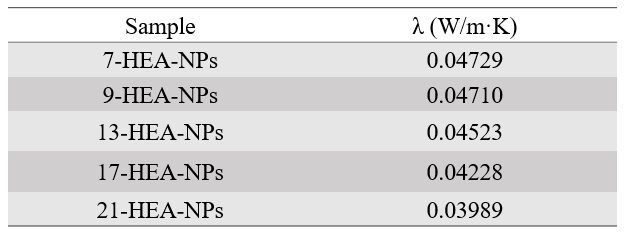


**Table S9.** Purity and particle diameters of the original metallic powders.


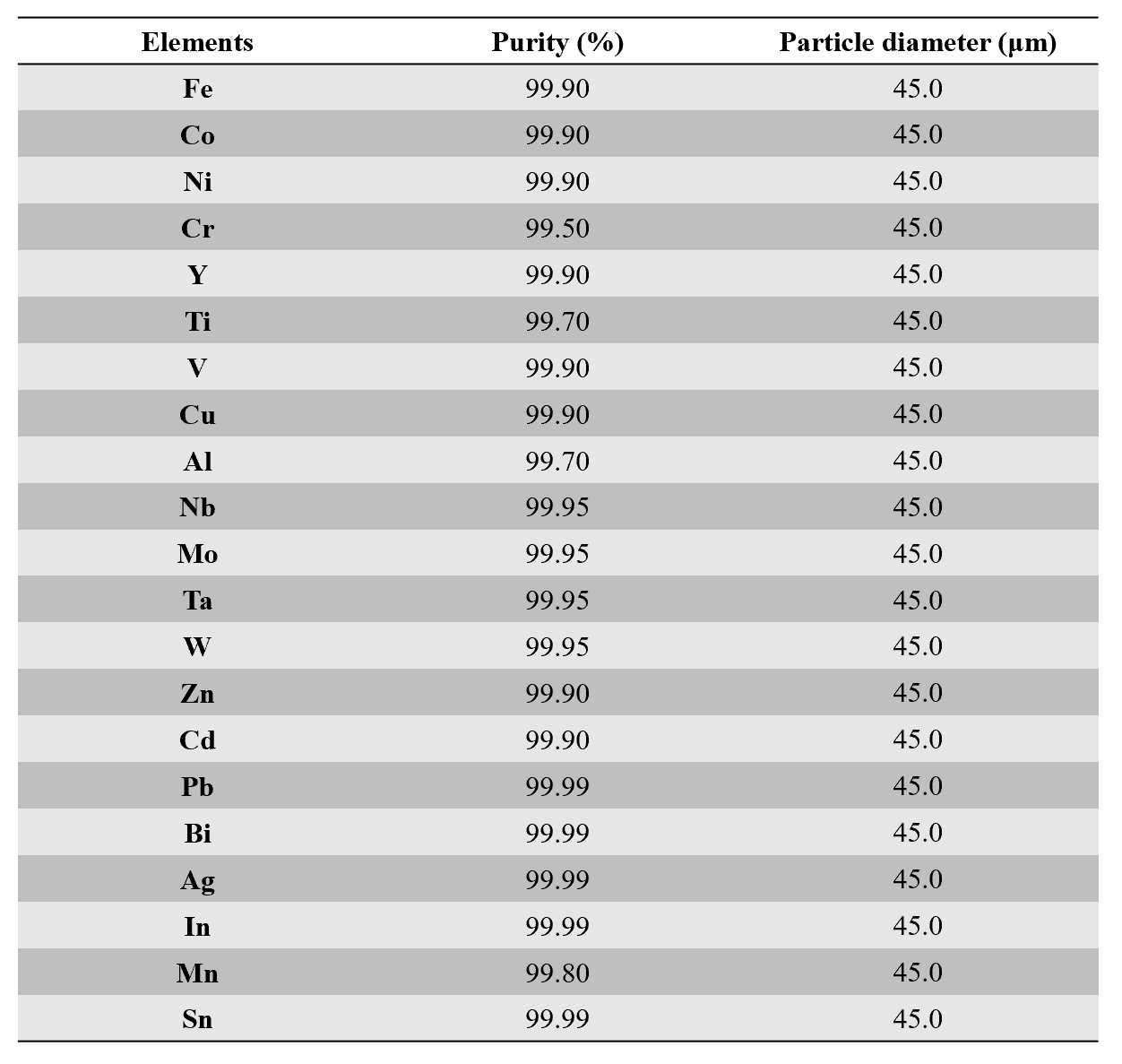


**Reference (S.M.)**

1. Guo S, Liu CT. Phase stability in high entropy alloys: Formation of solid-solution phase or amorphous phase. *Prog Nat Sci Mater Int* 2011; **21**: 433-46.

2. Troparevsky MC, Morris JR, Kent PRC*, et al.* Criteria for predicting the formation of single-phase high-entropy alloys. *Phys Rev X* 2015; **5**: 011041.

3. Yao Y, Huang Z, Xie P*, et al.* Carbothermal shock synthesis of high-entropy-alloy nanoparticles. *Science* 2018; **359**: 1489-94.

4. Senkov ON, Miracle DB, Chaput KJ*, et al.* Development and exploration of refractory high entropy alloys-a review. *J Mater Res* 2018; **33**: 3092-128.

5. Alcock C, Itkin V, Horrigan M. Vapour pressure equations for the metallic elements: 298–2500k. *Can Metall Q* 1984; **23**: 309-13.

6. Mimura K, Lim JW, Oh JM*, et al.* Refining effect of hydrogen plasma arc melting on titanium sponges. *Mater Lett* 2010; **64**: 411-4.

7. Skrabalak SE. Mashing up metals with carbothermal shock. *Science* 2018; **359**: 1467-.

8. Zhong G, Xu S, Dong Q*, et al.* Rapid, universal surface engineering of carbon materials via microwave-induced carbothermal shock. *Adv Funct Mater* 2021; **31**: 2010968.

9. Singh J, Benson GJCJoC. Measurement of the vapor pressure of methanol-n-decanol and ethanol-n-decanol mixtures. *Cana J Chem* 1968; **46**: 1249-54.

10. Morris J, Zellars GR. Vapor pressure of liquid copper and activities in liquid Fe-Cu alloys. *JOM* 1956; **8**: 1086-90.

11. Garg S, Bhatt Y, Sundaram C. Thermodynamic study of liquid Cu-Mg alloys by vapor pressure measurements. *Metall Trans* 1973; **4**: 283-9.

12. Yao Y, Huang Z, Hughes LA*, et al.* Extreme mixing in nanoscale transition metal alloys. *Matter* 2021; **4**: 2340-53.

13. Li R, Niu P, Yuan T*, et al.* Selective laser melting of an equiatomic cocrfemnni high-entropy alloy: Processability, non-equilibrium microstructure and mechanical property. *J Alloy Comp* 2018; **746**: 125-34.

14. Otto F, Yang Y, Bei H*, et al.* Relative effects of enthalpy and entropy on the phase stability of equiatomic high-entropy alloys. *Acta Mater* 2013; **61**: 2628-38.

15. Wang L, Zeng Z, Gao W*, et al.* Tunable intrinsic strain in two-dimensional transition metal electrocatalysts. *Science* 2019; **363**: 870-4.

16. Ahn M, Park Y, Lee SH*, et al.* Memristors based on (Zr, Hf, Nb, Ta, Mo, W) high-entropy oxides. *Adv Electron Mater* 2021; **7**: 2001258.

17. Xu X, Guo Y, Bloom BP*, et al.* Elemental core level shift in high entropy alloy nanoparticles via x-ray photoelectron spectroscopy analysis and first-principles calculation. *ACS Nano* 2020; **14**: 17704-12.

18. Yao R-Q, Zhou Y-T, Shi H*, et al.* Nanoporous surface high-entropy alloys as highly efficient multisite electrocatalysts for nonacidic hydrogen evolution reaction. *Adv Funct Mater* 2021; **31**: 2009613.

19. Stenzel D, Issac I, Wang K*, et al.* High entropy and low symmetry: Triclinic high-entropy molybdates. *Inorg Chem* 2021; **60**: 115-23.

20. Wang L, Mercier D, Zanna S*, et al.* Study of the surface oxides and corrosion behaviour of an equiatomic CoCrFeMnNi high entropy alloy by XPS and ToF-SIMS. *Corros Sci* 2020; **167**: 108507.

21. Zhao L, Yang Q, Guo W*, et al.* Co2.67s4-based photothermal membrane with high mechanical properties for efficient solar water evaporation and photothermal antibacterial applications. *ACS Appl Mater Interfaces* 2019; **11**: 20820-7.

22. Zhou L, Tan Y, Wang J*, et al.* 3D self-assembly of aluminium nanoparticles for plasmon-enhanced solar desalination. *Nat Photonics* 2016; **10**: 393-8.

23. Hu G, Cao Y, Huang M*, et al.* Salt-resistant carbon nanotubes/polyvinyl alcohol hybrid gels with tunable water transport for high-efficiency and long-term solar steam generation. *Energy Technology* 2020; **8**: 1900721.

24. Hu X, Xu W, Zhou L*, et al.* Tailoring graphene oxide-based aerogels for efficient solar steam generation under one sun. *Adv Mater* 2017; **29**: 1604031.

25. Li C, Jiang D, Huo B*, et al.* Scalable and robust bilayer polymer foams for highly efficient and stable solar desalination. *Nano Energy* 2019; **60**: 841-9.

26. Li J, Wang X, Lin Z*, et al.* Over 10 kg m^-2^ h^-1^ evaporation rate enabled by a 3D interconnected porous carbon foam. *Joule* 2020; **4**: 928-37.

27. Wang S, Almenabawy SM, Kherani NP*, et al.* Solar-driven interfacial water evaporation using open-porous pdms embedded with carbon nanoparticles. *ACS Appl Energy Mater* 2020; **3**: 3378-86.

28. Wang X, Li X, Liu G*, et al.* An interfacial solar heating assisted liquid sorbent atmospheric water generator. *Angew Chem Int Ed* 2019; **58**: 12054-8.

29. Wang Z, Yan Y, Shen X*, et al.* A wood-polypyrrole composite as a photothermal conversion device for solar evaporation enhancement. *J Mater Chem A* 2019; **7**: 20706-12.

30. Lei W, Khan S, Chen L*, et al.* Hierarchical structures hydrogel evaporator and superhydrophilic water collect device for efficient solar steam evaporation. *Nano Res* 2021; **14**: 1135-40.

31. Li W, Li X, Chang W*, et al.* Vertically aligned reduced graphene oxide/Ti_3_C_2_T_x_ mxene hybrid hydrogel for highly efficient solar steam generation. *Nano Res* 2020; **13**: 3048-56.

32. Shi L, Wang Y, Zhang L*, et al.* Rational design of a bi-layered reduced graphene oxide film on polystyrene foam for solar-driven interfacial water evaporation. *J Mater Chem A* 2017; **5**: 16212-9.

33. Yu K, Shao P, Meng P*, et al.* Superhydrophilic and highly elastic monolithic sponge for efficient solar-driven radioactive wastewater treatment under one sun. *J Hazard Mater* 2020; **392**: 122350.

34. Shen C, Zhu Y, Xiao X*, et al.* Economical salt-resistant superhydrophobic photothermal membrane for highly efficient and stable solar desalination. *ACS Appl Mater Interfaces* 2020; **12**: 35142-51.

35. Qi Q, Wang Y, Wang W*, et al.* High-efficiency solar evaporator prepared by one-step carbon nanotubes loading on cotton fabric toward water purification. *Sci Total Environ* 2020; **698**: 134136.

36. Mu P, Zhang Z, Bai W*, et al.* Superwetting monolithic hollow-carbon-nanotubes aerogels with hierarchically nanoporous structure for efficient solar steam generation. *Adv Energy Mater* 2019; **9**: 1802158.

37. Wu L, Dong Z, Cai Z*, et al.* Highly efficient three-dimensional solar evaporator for high salinity desalination by localized crystallization. *Nat Commun* 2020; **11**: 521.

38. Qiu P, Liu F, Xu C*, et al.* Porous three-dimensional carbon foams with interconnected microchannels for high-efficiency solar-to-vapor conversion and desalination. *J Mater Chem A* 2019; **7**: 13036-42.

39. Gong F, Li H, Wang W*, et al.* Scalable, eco-friendly and ultrafast solar steam generators based on one-step melamine-derived carbon sponges toward water purification. *Nano Energy* 2019; **58**: 322-30.

40. Wang C, Wang J, Li Z*, et al.* Superhydrophilic porous carbon foam as a self-desalting monolithic solar steam generation device with high energy efficiency. *J Mater Chem A* 2020; **8**: 9528-35.

41. Chen L, Zhao S, Hasi Q-M*, et al.* Porous carbon nanofoam derived from pitch as solar receiver for efficient solar steam generation. *Global Challenges* 2020; **4**: 1900098.

42. Yang F, Chen J, Ye Z*, et al.* Ni-based plasmonic/magnetic nanostructures as efficient light absorbers for steam generation. *Adv Funct Mater* 2021; **31**: 2006294.

43. Zheng Z, Li H, Zhang X*, et al.* High-absorption solar steam device comprising Au@Bi_2_MoO_6_-Cds: Extraordinary desalination and electricity generation. *Nano Energy* 2020; **68**: 104298.

44. Zhao L, Du C, Zhou C*, et al.* Structurally ordered agnps@C_3_N_4_/Go membranes toward solar-driven freshwater generation. *ACS Sustainable Chem Eng* 2020; **8**: 4362-70.

45. Zhang Q, Li L, Jiang B*, et al.* Flexible and mildew-resistant wood-derived aerogel for stable and efficient solar desalination. *ACS Appl Mater Interfaces* 2020; **12**: 28179-87.

46. Zhou L, Tan Y, Ji D*, et al.* Self-assembly of highly efficient, broadband plasmonic absorbers for solar steam generation. *Sci Adv* 2016; **2**: e1501227.

47. Li W, Tekell MC, Huang Y*, et al.* Synergistic high-rate solar steaming and mercury removal with MoS_2_/C @ polyurethane composite sponges. *Adv Energy Mater* 2018; **8**: 1802108.

48. Chen X, Meng C, Wang Y*, et al.* Laser-synthesized rutile tio_2_ with abundant oxygen vacancies for enhanced solar water evaporation. *ACS Sustainable Chem Eng* 2020; **8**: 1095-101.

49. Bian Y, Tang K, Xu Z*, et al.* Highly efficient solar steam generation by hybrid plasmonic structured tin/mesoporous anodized alumina membrane. *J Mater Res* 2018; **33**: 3857-69.

50. Wang S, Almenabawy SM, Kherani NP*, et al.* Solar-driven interfacial water evaporation using open-porous pdms embedded with carbon nanoparticles. *ACS Appl Energy Mater* 2020; **3**: 3378-86.

51. He J, Zhang Z, Xiao C*, et al.* High-performance salt-rejecting and cost-effective superhydrophilic porous monolithic polymer foam for solar steam generation. *ACS Appl Mater Interfaces* 2020; **12**: 16308-18.

52. Li W, Li Z, Bertelsmann K*, et al.* Portable low-pressure solar steaming-collection unisystem with polypyrrole origamis. *Adv Mater* 2019; **31**: 1900720.

53. Xiao C, Chen L, Mu P*, et al.* Sugarcane-based photothermal materials for efficient solar steam generation. *ChemistrySelect* 2019; **4**: 7891-5.
